# Fast and Accurate Bacterial Species Identification in Urine Specimens Using LC-MS/MS Mass Spectrometry and Machine Learning*[Fn FN2]

**DOI:** 10.1074/mcp.TIR119.001559

**Published:** 2019-10-04

**Authors:** Florence Roux-Dalvai, Clarisse Gotti, Mickaël Leclercq, Marie-Claude Hélie, Maurice Boissinot, Tabiwang N. Arrey, Claire Dauly, Frédéric Fournier, Isabelle Kelly, Judith Marcoux, Julie Bestman-Smith, Michel G. Bergeron, Arnaud Droit

**Affiliations:** ‡ Proteomics platform, CHU de Québec – Université Laval Research Center, Québec City, Québec, Canada; § Computational Biology Laboratory, CHU de Québec – Université Laval Research Center, Québec City, Québec, Canada; ¶ Département de Médecine Moléculaire, Faculté de médecine, Université Laval, Québec City, QC, Canada; ‖ Centre de Recherche en Infectiologie de l'Université Laval, Axe Maladies Infectieuses et Immunitaires, Centre de Recherche du CHU de Québec-Université Laval, Québec City, Canada; ** Département de microbiologie-infectiologie et d'immunologie, Faculté de médecine, Université Laval, Québec City, Québec, Canada; ‡‡ Thermo Fisher Scientific, Bremen, Germany; §§ Laboratoire de microbiologie-infectiologie, CHU de Québec-Université Laval, pavillon Hôpital de l'Enfant-Jésus, Québec City, Québec, Canada

**Keywords:** Microbiology, bacteria, SWATH-MS, tandem mass spectrometry, targeted mass spectrometry, urine analysis, LC-MS/MS, machine learning

## Abstract

Fast identification of microbial species in clinical samples is essential to provide an appropriate antibiotherapy to the patient and reduce the prescription of broad-spectrum antimicrobials leading to antibioresistances. MALDI-TOF-MS technology has become a tool of choice for microbial identification but has several drawbacks: it requires a long step of bacterial culture before analysis (≥24 h), has a low specificity and is not quantitative. We developed a new strategy for identifying bacterial species in urine using specific LC-MS/MS peptidic signatures. In the first training step, libraries of peptides are obtained on pure bacterial colonies in DDA mode, their detection in urine is then verified in DIA mode, followed by the use of machine learning classifiers (NaiveBayes, BayesNet and Hoeffding tree) to define a peptidic signature to distinguish each bacterial species from the others. Then, in the second step, this signature is monitored in unknown urine samples using targeted proteomics. This method, allowing bacterial identification in less than 4 h, has been applied to fifteen species representing 84% of all Urinary Tract Infections. More than 31,000 peptides in 190 samples were quantified by DIA and classified by machine learning to determine an 82 peptides signature and build a prediction model. This signature was validated for its use in routine using Parallel Reaction Monitoring on two different instruments. Linearity and reproducibility of the method were demonstrated as well as its accuracy on donor specimens. Within 4h and without bacterial culture, our method was able to predict the predominant bacteria infecting a sample in 97% of cases and 100% above the standard threshold. This work demonstrates the efficiency of our method for the rapid and specific identification of the bacterial species causing UTI and could be extended in the future to other biological specimens and to bacteria having specific virulence or resistance factors.

## Abstract (precis)

We have developed a new method for the identification of bacterial species causing Urinary Tract Infections. The first training step used DIA analysis on multiple replicates of bacterial inoculates to define a peptide signature by machine learning classifiers. In a second identification step, the signature is monitored by targeted proteomics on unknown samples. This fast, culture-free and accurate method paves the way of the development of new diagnostic approaches limiting the emergence of antimicrobial resistances.

## Graphical Abstract

**Figure:**
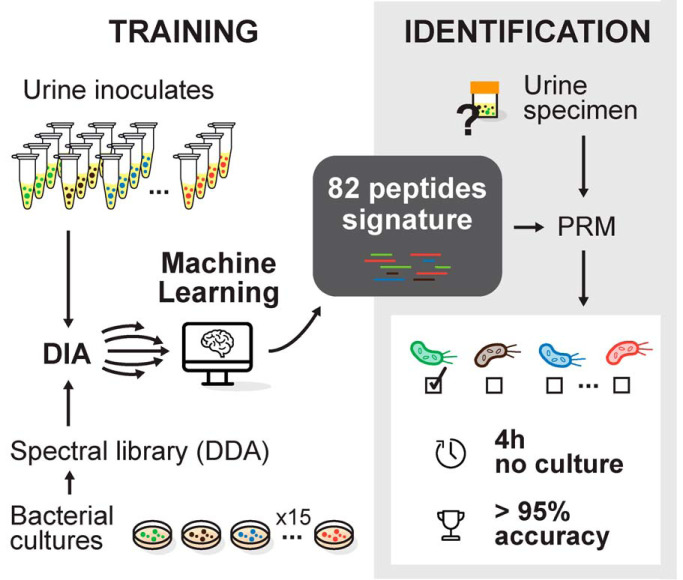

Highlights

- Fast and culture-free method for the identification of the 15 bacterial species causing UTIs.
- Combination of DIA analysis and machine learning algorithms to define a peptide signature.
- High accuracy, good linearity and reproducibility, sensitivity below standard threshold.
- Transferability to other laboratories and other mass spectrometers.

The identification of the bacterial species or strains present in a biological sample is essential in many fields of microbiology. Epidemiology, for instance, tracks the spreading of microorganisms related to infectious diseases; food safety laboratories ensure the distribution of pathogen-free products to the consumers; environmental bacteria have a strong impact on maintaining the equilibrium of ecosystems; and clinical laboratories require fast diagnosis methods to provide appropriate treatment to patients with a bacterial infection. However, standard methods for the identification of pathogens requires a time-consuming bacterial culture followed by another long step of immunological or biochemical tests of varying duration and cumbersomeness (1–3). During this period, typically of 24 to 48 h but could extend to weeks, patients received broad spectrum antimicrobial treatments. Although this strategy is efficient to release the infection for most cases, it is also known to have a strong impact on the development of antimicrobial resistances. Indeed, among patient urines tested for UTI, a large proportion are found not infected. For others, the prescription of broad-spectrum antibiotics, rather than species-specific antibiotics, might lower the efficiency of the therapy (4, 5). But in all cases, this misuse of antibiotics increases the emergence of multi-drug resistant bacteria (6–9). Therefore, there is a need for the development of fast and robust methods for bacterial identification, to improve therapies and guide rational use of antibiotics. Indeed, identification within few hours would allow to wait for the analysis result before initiating the treatment and then reduce the over-prescription of antibiotics to non-infected patients. But also, the knowledge of bacterial species could permit the early use of targeted narrow-spectrum antibiotics thus limiting the selection of resistant species in the overall population.

Genotyping methods, which are based on the sequencing of partial (16S small subunit ribosomal [rRNA] gene sequencing) or entire (Whole Genome Sequencing) genomes of the microorganisms contained in a sample, are promising because they do not require bacterial culture and can be applied to complex samples containing several species (10, 11). However, the cost and the time required to get identification by sequencing methods preclude their use in routine laboratories. In addition, if 16S rRNA sequencing can provide a quite rapid identification (typically 24 h), the high conservation of 16S gene sequences across bacterial families and species often limits the precision of identification to the genus level (12, 13). By contrast, Whole Genome Sequencing can provide an efficient species and even strain typing, but the cost and the time required to get the results is strongly extended by the sequencing itself and by the data analysis. Moreover, this analysis requires expert scientific knowledge to provide a confident genome assembly as well as large computing resources (14, 15).

In the past few years, Matrix-Assisted Laser Desorption Ionization - Time of Flight Mass Spectrometry (MALDI-TOF MS)^1^ analysis of microbial proteins has made a breakthrough in routine laboratories for bacterial identification (16–19). This fast, inexpensive, and automatable technology can replace the conventional phenotype-based methods, hence reducing the time required to get an identification from 2 or 4 days to less than 50 h. For those reasons, two mass spectrometers, the Biotyper (Bruker) and the Vitek-MS (Shimadzu-BioMérieux), have been approved for clinical use by health governmental organizations of most countries including the United States Food and Drug Administration (FDA) in 2013 (20). In the typical workflow, bacterial colonies isolated by culture are submitted to a fast sample preparation (typically, a treatment with formic acid and ethanol) before acquisition of protein mass spectra that are used to interrogate a spectral database providing a confidence score for the bacterium identification, an information a physician can use to diagnose the infection.

Despite its numerous advantages, bacterial identification by MALDI-TOF MS has several drawbacks: 1) it requires a lengthy culture step to isolate bacterial colonies, because the detection is based on a comparison with spectral database acquired on pure colonies. For the same reason, it is not able to identify polymicrobial infections (*i.e.*: when several species are present in the same sample) without analyzing several types of colonies visually selected on the culture plate; 2) because of the minimal sample preparation, the information contained in the spectra is restricted to the most abundant molecules, thus limiting the specificity of the method and its capability to identify certain species or subspecies and; 3) it is not quantitative, a potentially important information for certain specimens where pathogens need to be distinguished from the normal microbiota, or when a certain level of infection needs to be reached to necessitate antibiotherapy.

To overcome the above-mentioned issues, several studies have tried to improve MALDI-TOF bacterial identification (21). For instance, Clark and colleagues refined the specificity of the method to identify *Escherichia coli* pathotypes by examining specific peaks in the spectra (22). Other investigators have tried to improve the specificity using trypsin digestion which allows the accession to a larger set of molecules and the generation of a Peptide Mass Fingerprint of the bacterial subspecies (23). Several studies skip the culture step to provide a faster identification, especially in the case of sepsis where MALDI-TOF acquisition is performed directly from a positive blood culture sample (24, 25). However, it has been shown that sample preparation methods, which are not homogenous from lab to lab, can influence the rate of correct identification of certain microorganisms (26).

Although these studies could improve the standard workflow, they are limited by the sensitivity and the specificity of MALDI-TOF mass spectrometer. Therefore, recent studies have investigated the possibility of using LC-MS (Liquid Chromatography - Mass Spectrometry) methods which, because of their high sensitivity and specificity, have replaced MALDI-TOF MS in most research laboratories. Wang and colleagues used the LC-MS approach to identify biomarkers of five major bacterial species in bronchoalveolar lavage specimen (27) and performed strain typing for *Acinobacter baumanii* (28), Karlsson R et *al.* used it for proteotyping within the mitis group of *Streptococcus* genus (29) and Cheng *et al.* also used LC-MS/MS in Selected Reaction Monitoring (SRM) mode to target specific peptides of the flagella to type *Escherichia coli* at strain level (30). Bioinformatics tools have also been developed to help in the identification of bacteria from “bottom up” proteomics data (*i.e.* trypsin-digested proteins). These methods were able to reach 89 to 98.5% correct classification rates at the species level but these values have only been demonstrated after a step of bacterial growth (31, 32).

Taking the advantages of sensitivity and specificity from nanoscale LC-MS/MS technology, and based on these previous studies, we developed a new pipeline using modern proteomics (DIA - Data Independent Acquisition mode) and machine learning algorithms to identify biomarkers able to speciate a set of bacteria of interest in urine specimens. This strategy is based on two steps (Fig. 1): 1) a training step, that enables to define a peptidic signature for the bacteria of interest and 2) an identification step where the signature is monitored by targeted proteomics to get the identification of bacteria in the infected samples.

Once the training step has been developed, the second step can be performed in routine laboratories on multiple samples and with any type of mass spectrometer working in PRM (Parallel Reaction Monitoring) or SRM (Selected Reaction Monitoring) modes.

This pipeline has been applied to the 15 bacterial species most frequently found in Urinary Tract Infections (UTI). Indeed, urine is the most common clinical specimen with hundreds of samples analyzed each day in most clinical laboratories. Moreover, UTI is one of the most frequent types of infection in humans: it has been demonstrated that 50 to 60% of women in western countries will have at least one UTI in their lifetime (33). As reported by statistics of the Enfant-Jésus hospital in Québec City, which analyzes 300 urine specimens each day on average, 68.2% of these samples are infected by the same 4 bacterial species (*Escherichia coli, Streptococcus agalactiae, Klebsiella pneumoniae* and *Enterococcus faecalis*) and 15 species are responsible for more than 84% of all UTI (supplemental Fig. S1). According to literature reports, these are the most frequently found species in UTI (33, 34).

Our original method enables to define a peptidic signature which, when monitored by targeted proteomics, can detect which of the 15 bacterial species is present in the urine sample, in less than 4 h, without any bacterial culture. We also demonstrated that the peptidic signature is transferable to other laboratories and to other mass spectrometers. In addition, we compared the efficiency of our method to the MALDI-TOF standard workflow.

## EXPERIMENTAL PROCEDURES

### Bacterial Culture and Counting

Bacterial strains were obtained from the Culture Collection of Centre de Recherche en Infectiologie of Université Laval (CCRI, Québec, Canada).

The bacterial strains used and their corresponding culture conditions are listed in supplementary methods. Semi-log broth bacterial culture calibrated to 0.5 MacFarland suspension were prepared and used for spectral libraries generation or for urine inoculation. In parallel, they were counted by incubation of 100 μl of serial dilutions on blood agar plate (Supplementary Methods).

### Urine Collection and Bacterial Inoculation

To mimic urinary tract infections, 1 to 200 μl of semi-log broth culture suspension, corresponding to an estimated final amount of 1 × 10^4^ to 1 × 10^6^ CFU/ml, were spiked into 10 ml of urine obtained from six different healthy volunteers. The exact concentration of the inoculated cultures was determined in parallel by culture on agar plates as described above.

Moreover, urine specimens from 27 patients were collected at the Microbiology-Infectiology laboratory of Enfant-Jésus hospital of CHU de Québec (Québec, Canada) after few microliters had been used for standard MALDI-TOF analysis. The specimens were kept on ice during the transportation (< 1 h) and used immediately.

The consent of all donors was obtained as described in the ethical approval of the Comité d'Éthique de la Recherche of CHU de Québec - Université Laval (recording number 2016–2656).

### Sample Preparation for Spectral Libraries

For the generation of bacterial spectral libraries, bacteria from 1 ml of semi-log broth bacterial cultures were pelleted by centrifugation at 10,000 × *g* for 15 min, the supernatant was discarded and the pellet was washed three times with 1 ml of 50 mm Tris and centrifuged in the same conditions. The final pellet was frozen dried and stored at −20 °C.

Pellets were then resuspended with 50 mm of ammonium bicarbonate and 600 units of mutanolysin (Sigma-Aldrich, cat no. M9901) were added to help bacterial lysis by digestion of cell wall peptidoglycan. After a 1-hour incubation at 37 °C, 0.5% sodium deoxycholate (SDC) and 20 mm dithiothreitol (DTT) (final concentrations) were added and bacterial inactivation was performed by heating 10 min at 95 °C. Lysis was achieved by sonication for 15 min with a Bioruptor® system (Diagenode), with cycles of 30 s ON/30 s OFF, high level. A final centrifugation at 16,000 × *g* during 15 min was performed to remove cell debris, and protein concentration in the supernatant was measured using a Bradford assay.

Before proteolytic digestion, SDC concentration was adjusted to 1% and 120 μg of proteins from each bacterial culture were digested by the addition of trypsin (Promega) in a 1:50 (enzyme/protein) ratio, during 1 h at 58 °C. Trypsin reaction was then stopped by acidification with 350 μl of 5% formic acid (FA), which also leads to precipitation of the SDC. After centrifugation at 16,000 × *g* for 5 min, the supernatant was collected, the peptides were purified on Oasis HLB cartridge 10 mg (Waters) and vacuum-dried.

The pellet was resuspended in 10 mm Ammonium bicarbonate pH10 and an equivalent of 110 μg of peptides were fractionated on an Agilent 1200 Series System HPLC equipped with Agilent extend C_18_ (1.0 mm × 150 mm, 3.5 μm) column. Peptides were loaded at 1 ml/min of solvent A (10 mm ammonium bicarbonate pH10) and eluted by the addition of solvent B (90% acetonitrile, 10% ammonium bicarbonate pH10) with a gradient 5 to 35% solvent B during 60 min and 35 to 70% solvent B during 24 min. Fractions were collected in a 96 well plates at 1 min intervals and finally pooled in rows into 8 fractions which were vacuum-dried.

Each fraction was resuspended in 2% acetonitrile (ACN)/0.05% trifluoroacetic acid (TFA) at 0.2 μg/μl and 1X iRT peptides (Biognosys) were added. An equivalent of 1 μg of peptides was injected on LC-MS/MS system for each fraction of each bacterial species.

### Preparation of Urine Samples

Urine specimens (10 ml), either from patients or artificially inoculated from healthy urine, were treated the same way: human cells were initially pelleted by low speed centrifugation for 5 min at 1000 × *g*, and the supernatant was high speed centrifuged for 15 min at 10,000 × *g* to collect bacteria. Bacterial pellets were then washed with 1 ml of 50 mm Tris and centrifuged again in the same conditions, another cycle of wash and centrifugation was added, and the resulting pellet was frozen dried.

Protocols for protein extraction, trypsin digestion and peptide purification are described above in the Sample Preparation for Spectral Libraries section and were modified as follows: for each sample, 50 units of mutanolysin was used, 250 ng of trypsin was added for the digestion which was then stopped with 1 μl of 100% FA and peptides were purified with StageTips (35) containing C18 reverse phase (3 m Empore C18 Extraction Disks). Samples were resuspended in 10 μl of 2% ACN, 0.05% TFA and 1X iRT peptides (Biognosys) were added. Half of the final volume was injected on LC-MS/MS system.

### LC-MS/MS Acquisitions

Samples were analyzed by nanoLC/MS using a UltiMate^TM^ 3000 NanoRSLC system (Thermo Fisher Scientific Germering, Germany) coupled to an Orbitrap Fusion Tribrid mass spectrometer with ETD option (Thermo Fisher Scientific, San Jose, CA, Instrument Control Software version 2.0) installed in CHU de Québec - Université Laval research center (Québec, Canada). Peptides were trapped at 20 μl/min in loading solvent (2% acetonitrile, 0.05% TFA) on a μ-Precolumn, 300 μm i.d × 5 mm, C18 PepMap100, 5 μm, 100Å (Thermo Fisher Scientific) for 5 min. Then, the pre-column was switched online with a PepMap100 RSLC, C18 3 μm, 100Å, 75 μm i.d. × 50 cm column (Thermo Fisher Scientific) and the peptides were eluted with a linear gradient from 5–40% solvent B (A: 0.1% formic acid, B: 80% acetonitrile in 0.1% formic acid) in 90 min, at 300 nL/min flow rate.

For faster measurements, a Q Exactive HF-X mass spectrometer (Thermo Fisher Scientific, Bremen, Germany, Instrument Control Software version 2.9) was coupled to an UltiMate^TM^ 3000 RSLCnano system (Thermo Fisher Scientific, Germering, Germany) operated in capillary flow chromatography (installed in Thermo Fisher Scientific mass spectrometers factory in Bremen, Germany). Peptides were loaded onto a μ-Precolumn, 300 μm i.d × 5 mm, C18 PepMap100, 5 μm, 100Å (Thermo Fisher Scientific) at a flow rate of 50 μl/min, loading solvent (2% ACN, 0.05% TFA) for 1 min. Then, the pre-column was switched online with a PepMap100 RSLC, C18 2 μm, 100Å, 150 μm i.d. × 15 cm column (Thermo Fisher Scientific). The peptides were eluted with a linear gradient from 6–60% solvent B (A: 0.1% formic acid, B: 80% acetonitrile in 0.1% formic acid) in 34 min, at 1 μl/min flow rate. Mass spectrometer parameters settings in DDA, DIA and PRM modes on both instruments are described in the Supplementary methods.

### Peptides Libraries Generation

Proteome Discoverer 2.1.0.81 software (Thermo Fisher Scientific) was used to search DDA raw files against Uniprot bacterial databases (databases are listed in Supplementary Methods). Peak lists were generated with the Spectrum Selector node (default parameters) of Proteome Discoverer and searched using Mascot search engine version 2.5 (MatrixScience). Parameters were set for trypsin enzyme digestion specificity with two possible missed cleavages, methionine oxidation, asparagine and glutamine deamidation were set as variable modifications, and mass search tolerance were 10 ppm and 0.6 Da for MS and MS/MS respectively. Peptides were then validated based on target/decoy search using Percolator software with a Delta Cn parameter >0.05 for PSM filtering (36). Only high confidence peptides (FDR<1% at peptide level) were finally considered .

### Peptides Selection and Signal Extraction in DIA Analyses

For higher confidence and reproducibility in peptide identification, DIA signal extraction was performed on a selected part of the peptides identified in spectral libraries. Only peptides without missed cleavage or potential missed cleavage, having at least 8 amino acids in their sequence without any methionine and cysteine and identified in at least 6 peptide spectrum matches (PSM) were considered. The list of peptides from the 15 bacterial species was then searched with the Unipept software (37, 38) to delete peptide sequences also found in the human proteome, and to associate each peptide to the bacterial proteome it belongs to. Finally, for each bacterium separately, a list of potentially observable peptides was built and imported into Skyline 4.1.0.11796 (39, 40). Shuffle decoy peptides were added to allow further scoring. A spectral library common to the 15 bacterial species was generated with the BiblioSpec 2.0 tool implemented into Skyline using the Mascot .dat files generated from all the individual DDA analyses and a 0.95 cut-off score (Skyline default value) on the Mascot expected value (homology threshold). Retention time predictor was used considering the iRT peptides retention time values. Orbitrap resolving power was set at 30 K at 200 *m*/*z* with a high selectivity extraction. For each precursor (2+ or 3+), only 6 fragments (b or y) were automatically selected within 10 min around the predicted RT and their corresponding signal was extracted from the raw files, the signal of precursor masses was not extracted. mProphet algorithm (41) was used within Skyline to score the peaks, considering the decoys and the second-best peaks.

Only peaks with a Skyline dot product (dotP) > 0.75 and a *q*-value < 0.01 were considered as quantifiable and for each of them, the peptide areas (*i.e.* the sum of the areas under the curve of the 6 most intense fragments) were normalized with the sum of the 10 iRT peptides. The non-quantifiable peaks received a value of 0. Finally, only the best intensity precursor of each peptide was kept to build a final list of peptides with their corresponding area values.

### Machine Learning

We applied various machine learning models and several feature search approaches to identify a peptidic signature using BioDiscML (42), a tool based on Weka Java library (43).

Briefly, BioDiscML works as follows: during the loading of input data, a sampling is performed to create a test set not used during learning. From the training set, the features, here the peptides, are identified and ranked by their predictive power through information gain ranking for classification. Then, optimal signatures are built using a combination of various stepwise feature selection overall input features and model search approaches. For each iteration on all best ranked features, BioDiscML runs a set of stepwise methods (forward, backward, or a combination of both) using many machine learning classifiers (*e.g.* Naïve Bayes, Random Forest) that are evaluated by cross validation procedures (*e.g.* k-fold, Bootstrapping, repeated holdout, evaluation on test set) and on the test set.

### Peptidic Signature Validation and Bacterial Identification Prediction

After PRM analysis using the Orbitrap Fusion or the Q Exactive HF-X instrument, the Skyline software 4.1.0.11796 (39, 40) was used to extract the signal of the 82 peptides signature (*i.e.* the sum of the areas under the curve of the 6 most intense fragments) in each sample. The peptides were considered as detected if they met the following Skyline criteria: dotp > 0.85 and average mass error < 10 ppm, or dotp > 0.75 and average mass error < 3 ppm. For each analysis (inoculated urines or patient sample), the list of detected peptides was submitted to the Bayesian Network model trained in the previous section for prediction purposes.

### MALDI-TOF Analysis

For all MALDI-TOF analyses, the standard procedure of the Enfant-Jésus hospital microbiology laboratory was used. Briefly, 1 μl of urine was streaked on blood agar plate and 1 μl on Mc Conkey agar plates (Oxoid). The plates were incubated for 18 h at 35 °C. Isolated colonies with homogenous aspect were selected for MS analysis. The non-treated colonies were spotted on MALDI plate with HCCA matrix. MALDI-TOF MS analysis was performed on a Bruker Biotyper instrument using the Flux control version 3.4 (build 135) software and 7311 MSPs database.

### Experimental Design and Statistical Rationale

To obtain a high quality peptidic signature using machine learning algorithms, 9 high-level and 3 low-level inoculations replicates of each bacterial species were used. Ten non-inoculated urine specimens (biological replicates) were used as control. For the validation of the method in targeted proteomics (Tier 3 level), four different biological replicates of each bacterial inoculation in urine were monitored in two different analysis conditions. The four non-inoculated urines were used as control. Finally, urine from 27 different patients were used to compare the method to conventional MALDI-TOF analysis. Prediction accuracies were reported.

## RESULTS

**Fig. 1.**
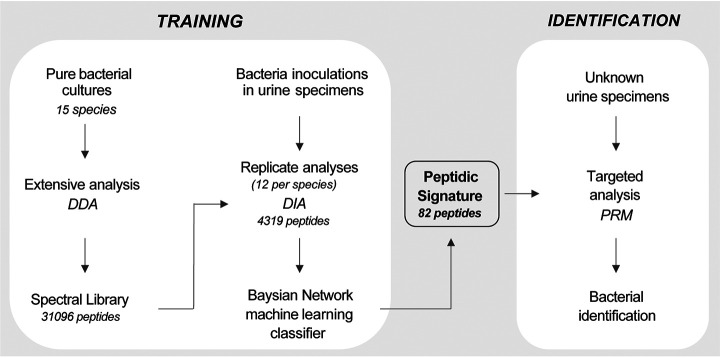
**Workflow of the method for bacterial identification.** The workflow is composed of two steps: the “training” step defines of a peptidic signature for the bacteria of interest; the “identification” step uses this signature in routine to identify bacteria in biological samples.

Our workflow for bacterial identification is composed of two steps: 1) a training step which includes the LC-MSMS acquisition of a peptide library on pure bacterial colonies in Data Dependent Acquisition mode followed by Data Independent Acquisition analyses to obtain information on bacterial peptides observability in urine and the generation of a short peptidic signature by machine learning models and 2) an identification step where the signature is monitored in unknown samples by PRM to obtain a bacterial identification through a prediction algorithm (Fig. 1).

For the training step, to detect minor bacterial peptidic signals in the human proteic background, we used DIA acquisition, on an Orbitrap Fusion instrument operating in nanoflow rate, because of its high sensitivity and its ability to provide a deep coverage of bacterial proteomes by acquisition of all peptides contained in the sample (44, 45). Indeed, in contrast to DDA which uses a full scan MS for the detection of peptide species, the DIA mode, by systematic acquisition of small size windows all along the mass range, improves the dynamic range and, thus, the sensitivity of the analysis. However, the simultaneous fragmentation of peptides inside this small window generates a complex spectrum which cannot be searched with conventional database search engines.

### Acquisition of Bacteria Spectral Libraries

One of the proposed approaches to extract information from the DIA complex spectra is to use spectral libraries previously acquired in DDA mode on the same type of sample and annotated with peptide/protein identifications through a protein database search (44). In our study, we have generated these spectral libraries from pure bacterial colonies to be as exhaustive as possible and cover a very wide range of bacterial tryptic peptides, and subsequently be able to extract this specific bacterial peptide information from the DIA complex spectra contaminated with human biological material.

We have generated spectral libraries for the 15 bacterial species of interest. To do so, each species was cultivated separately, proteins were extracted and digested with trypsin as described in the Experimental Procedures section. The resulting peptides were fractionated by high-pH reversed phase chromatography. For each bacterial species, eight fractions were injected by LC-MS/MS in DDA mode and analyzed through a standard database search pipeline allowing the identification of 10686 to 29558 peptides at 1% FDR corresponding to 810 to 2438 protein groups (supplemental Tables S1 and S2). As anticipated based on their genome size, Gram-positive bacteria generated less protein identifications than the Gram-negative. Indeed, there was a good correlation between the number of proteins identified in our study and the genome length (Pearson correlation coefficient *r* = 0.82) or the protein count predicted from genomic data (Pearson correlation coefficient *r* = 0.83) of all those 15 species. Thus, peptide fractionation combined to mass spectrometry analysis on a high resolution and high sensitivity instrument allowed us to cover 22.3 to 48.4% of the Uniprot reference proteome of each of the 15 species (supplemental Table S1). Then, the whole list of peptide identifications was refined to filter out: 1) the peptides which may not to be reproducible from run to run (*i.e.* cysteine and methionine containing peptides, those containing trypsin missed cleavages, peptides shorter than eight amino-acids), and 2) the less abundant or less ionizable peptides (*i.e.* those having less than six peptide spectrum matches). Finally, we obtained a set of 31096 peptides which, according to their taxonomic affiliations, demonstrated a high redundancy across the 15 species (supplemental Fig. S2*A*).

This redundancy associated to our reproducibility filters showed that it is not possible to select from these data one or several specific peptides for each bacterial species that could would be further able to specifically sign for the presence of each distinct species in the urine. Indeed, not enough specific peptides are available when working which this large number of bacteria (*i.e.* 15) (supplemental Fig. S2*B* and S2*C*).

Thus, we aim to define a set of peptides that could be shared by several species, but which, taken together, form a pattern for each bacterial species to be identified. To obtain this 'peptidic signature' our strategy was to use deep proteome coverage combined to machine learning algorithms.

### Data Independent Analysis of Artificially Inoculated Urine Replicates

To define a peptidic signature of 15 bacterial species in the human urine background, we have generated 12 artificial sample replicates, for each species of our selection, by inoculating urine from healthy volunteers with bacterial culture. Two concentration levels were used set at 1 × 10^6^ CFU/ml (Colony Forming Unit per milliliter of urine) (*n* = 9) (high level) and below 1 × 10^5^ CFU/ml (*n* = 3) (low level) approximatively which corresponds to the threshold used by most clinical laboratories for considering a UTI requiring an antibiotherapy. A total of 190 samples were produced, including “blank” samples corresponding to non-inoculated urine as control. After protein extraction and short trypsin digestion, the resulting peptides were analyzed by LC-MS/MS in DIA mode. Compared to the DDA, DIA analysis enables a deep proteome coverage by reduction of the spectral dynamic range resulting in fewer missing values (46). However, MS/MS spectra acquired in DIA mode are the sum of fragments generated by all precursor peptides selected in the same DIA window. This yields complex spectra where peptides sequences can be deduced by extraction of their specific fragments contained in the spectral libraries previously generated on pure bacterial colonies as described above. To do so, we have used the Skyline software (39, 40) and the list of 31,096 selected peptides was used. An additional step of refinement was done to establish, for each species, the list of bacterial peptides to be searched for in the DIA runs. To this purpose, we used the Unipept software (37, 38) that enables to match peptide sequences with all matching taxa in UniProtKB databases. Starting from the non-redundant list of all peptides identified from the bacteria (31,096 peptides), Unipept was used to confirm in which of the 15 bacterial species these could theoretically be found. Indeed, because of the stochastic effect of DDA used for library generation, it might be that some peptides belonging to several species had been sequenced by MS/MS in only a subset of them. The Unipept software also helped us to remove peptide sequences shared with the human proteome (57 peptides), hence generating high confidence lists of expressed peptides for each of the 15 species, free of potential human interfering compounds (supplemental Table S3).

These lists and the corresponding spectra were added to Skyline for extracting DIA signals in the 12 replicates of each of the 15 inoculated samples. As retention time calibration peptides (iRT, Biognosys) were added in the DDA and DIA runs (performed with 90 min gradients), predicted RT could be used for signal extraction in a small window of 10 min, thus limiting the probability for the software to select background peaks. A list of decoy peptides generated by Skyline were also extracted in the same conditions to ensure the calculation of a scoring *q*-value through the mProphet algorithm (41) included in Skyline.

Finally, the peptides were considered as detected if they met the following criteria: mProphet *q*-value < 0.01 and library dot product (dotp) > 0.75. After normalization of the peptide ions area values (*i.e.* the sum of the 6 most intense fragments areas) by the sum of the iRT peptides area values and filtering for the best intensity precursor (when both doubly and triply-charged precursors were detected for the same peptide), the 15 final lists were combined into one, composed of 4319 peptides, (supplemental Table S4) and submitted to machine learning algorithms to classify the bacteria and identify a short peptidic signature. This computational method has been chosen for its ability to handle large data sets and to perform predictions on them using accurate statistical models (47).

### Peptidic Signature Generation by Machine Learning

In our study, before training classifiers, the dimension of the list of peptides was reduced by mutual information filter (*i.e.* Information Gain ranking), which ended with a list of first 1000 best peptides according to their ranking. After training and evaluation by BioDiscML, the peptidic signature was composed of feature subsets found by three models having very high predictive performance (AUC > 98% on test set): 1) 68 features signature found by forward stepwise feature selection optimized by Matthew's correlation coefficient criterion (48) using Naive Bayes classifier (49) with discretization parameter option, 2) 78 features signature found by forward stepwise feature selection optimized by Area Under the Curve (AUC) criterion using Bayesian Network classifier (50) with AD-Tree parameter option, and 3) 20 features signature found by forward stepwise feature selection combined with backward stepwise feature elimination optimized by Matthew's correlation coefficient criterion using Hoeffding Tree classifier (51) with default parameters. Because stepwise feature selection tends to remove all correlated features, we retrieved those using Pearson and Spearman correlations having >99% correlation. The choice of keeping the features and correlated features selected by more than one classifier was motivated by the need to have the largest and the most precise signature exempt of noisy features. Having highly correlated features here also mean preserving “backup” peptides in case of missing peptides (for instance at low bacterial concentrations) and thus improve the sensitivity threshold of the method. The overlap between the three signatures was 10 peptides (supplemental Fig. S3*A* and supplemental Table S5).

The obtained feature subsets of the three models were then merged into a list of 106 unique peptides which were manually curated by inspection into Skyline software. Peptides which show uncertain peak picking, those also found in blank samples, as well as pairs of peptides having the same precursor mass because of leucine/isoleucine amino-acids were deleted to obtain a final curated signature, composed of 82 peptides. Eight peptides from this list were observed in all three models (supplemental Fig. S3*B* and supplemental Table S5). The intensity values for this 82 peptides signature were then discretized into presence (intensity > 0) or no presence (intensity = 0) of a signal and was used to train a final Bayesian Network prediction model by automated learning. All new samples were analyzed using this predictive model. This model, trained on only high levels of concentration provided 100% classification accuracy on several *k*-fold cross-validations (*k* = 2, 5, 10) and was able to classify at 84% overall accuracy the low-level concentration samples (3 replicates per bacteria) corresponding to a concentration below the clinical threshold of 1 × 10^5^ CFU/ml.

**Fig. 2.**
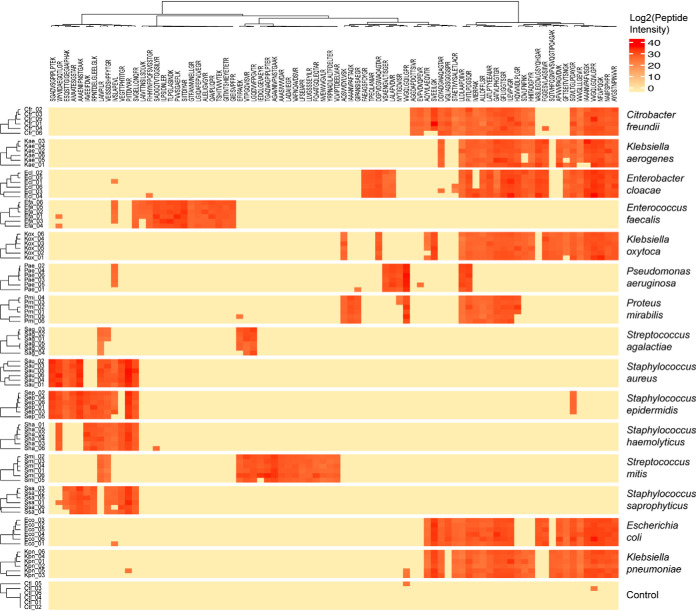
**Heatmap of the peptidic signature corresponding to the 15 most frequently found bacteria in UTI.** Intensity of each of the 82 peptides identified by the machine learning algorithm is represented for the six high-level concentration replicates of urine inoculation for each bacteria of interest. Data are presented with hierarchical clustering in rows and columns.

In the final signature, 5 to 26 peptides are observable for each bacterium (Fig. 2 and supplemental Fig. S4). Even though closely related species, such as *Streptococcus epidermidis* and *Staphylococcus aureus*, or *Klebsiella pneumoniae* and *Escherichia coli*, share up to 75% of common peptides there are always a few peptides to distinguish between them (4 and 7 peptides respectively in these two cases). For some very low concentration replicates, a few peptides, found high concentration replicates, were not detected. This loss affected the ability of the algorithm to predict the bacteria in only 15% of the tested low concentration replicates. Inversely, some false positive peptide detections were also observed, probably because of peak picking errors by Skyline in DIA runs, but they did not interfere with the bacterial prediction, assessing the robustness of the Bayesian Network model. As expected, most of the peptides composing the signature belong to relatively abundant proteins such as ribosomal proteins (*e.g.* 50S ribosomal protein L10, 30S ribosomal protein S5) or enzymes involved in amino acid metabolism (*e.g.* formate acetyltransferase) and glycolysis (*e.g.* GAPDH, pyruvate kinase) (52).

### Validation of the Signature by Targeted Proteomics

Because the machine learning algorithm has identified a short list of peptides allowing the discrimination of the 15 bacteria of interest, this list can now be monitored by targeted proteomics which is known to give a better reproducibility of measurements and a better sensitivity in peptide detection and could thus improve the limit of detection of bacterial species in urine (supplemental Table S6). The information on presence or absence of each of the 82 peptides of the signature is then given to the developed prediction model to obtain a probability of contamination. This step corresponds to the Identification step of our pipeline (Fig. 1). For this purpose, any type of mass spectrometer designed to perform targeted proteomics in Selected Reaction Monitoring (SRM) or Parallel Reaction Monitoring (PRM) modes can be used.

To validate our peptidic signature, we have initially used an Orbitrap Fusion Tribrid instrument in PRM mode to monitor precursor masses of the 82 peptides signature on samples resulting from inoculated urines. For this purpose, the four most frequently found bacteria in UTIs (*Escherichia coli, Streptococcus agalactiae, Enterococcus faecalis* and *Klebsiella pneumonia*) were inoculated at 5 different concentrations (from 2.56 × 10^4^ to 8.77 × 10^6^ CFU/ml) in urine from four different healthy volunteers (Supplemental Table 7). The samples were processed as described in the Experimental Procedures section and analyzed with a 90-min gradient typically used in research laboratories. Only half of the volume of each sample was injected whereas the other half was kept for validation on other instrument types as further described.

**Fig. 3.**
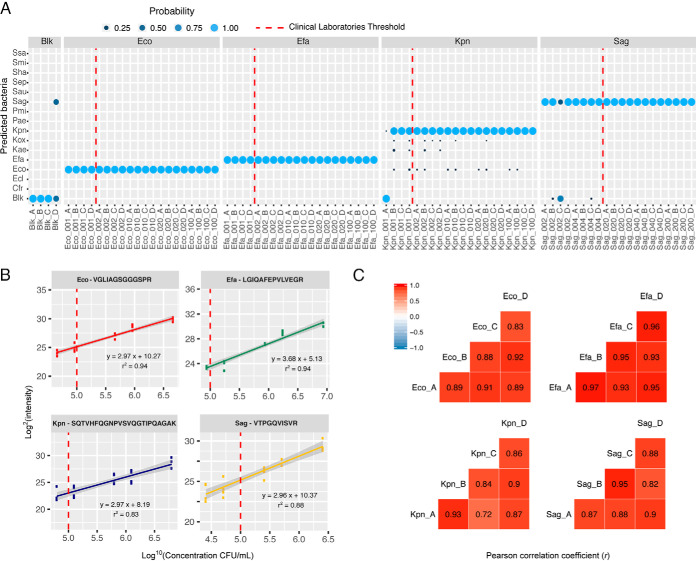
**Accuracy, linearity and reproducibility of the 'identification' step of the method performed on the four most frequent bacteria in UTI.** *A*, Prediction reported by the algorithm after peptidic signature monitoring by PRM associated with its probability (light blue : high probability, dark blue : low probability) for five concentrations corresponding to five inoculation volumes (1, 2, 10, 20 and 100 μl or 2, 4, 20, 40 and 200 μl) of four bacteria (Eco, Efa, Kpn or Sag) in urine of four different healthy volunteers (A, B, C, and D), dotted red line corresponds to the commonly used clinical laboratories detection threshold of 1 × 10^5^ CFU/ml; *B*, Linearity curves obtained for 4 peptides of the peptidic signature with the samples across the five tested concentrations, dotted red line corresponds to the commonly used clinical laboratories detection threshold of 1 × 10^5^ CFU/ml; *C*, Pearson correlation coefficients between two of the four biological samples (*i.e.* four different urines of healthy volunteers).

To validate the detection or non-detection of each peptide of the signature, the Skyline software was used associated with filtering criteria. Several criteria and values were tested to define the best filtering for the detection of these known samples (supplemental Table S8). The criteria giving the lower level of wrong prediction (dotP > 0.85 & ppm < 10 or dotP > 0.75 & ppm < 3) was retained and further reapplied for all unknown samples. Finally, the list of detected peptides for each replicate sample was submitted to the Bayesian Network model for evaluation. The probabilities of bacterial identification are shown on Fig. 3a and supplemental Table S9*A*. In 97% of the cases, the method was able to predict the correct species inoculated in the sample. We have investigated the factors which causes the wrong predictions in our data set. In most cases, this has been observed on urines samples inoculated at low bacterial concentrations (below the clinical laboratory threshold of 1e5 CFU/ml). In those cases, the signal of some peptides appears to be very low (or undetectable) and Skyline picks a wrong peak. These peaks are then filtered out by our criteria on dotp and mass shift. In all cases but one, the wrong prediction is reported as a “blank” (*i.e.* no contamination). Finally, when looking at the data above the 1 × 10^5^ CFU/ml threshold commonly used by clinical laboratories, the accuracy in prediction reaches 100%.

Using the intensities given by Skyline for each peptide, linearity curves have been plotted for each detected peptide of the signature (Fig. 3*B* and supplemental Fig. S5*A*–S5*D*). The median of determination coefficient (R^2^) over the 5 concentrations was 0.841, suggesting that the method could be used for the quantification of bacterial contamination in urine samples. Because four biological replicates (*i.e.* bacterial inoculation in urines coming from four different volunteers) have been analyzed for each bacteria concentration, the reproducibility of the method was evaluated. Scatter plots and Pearson correlation factors were calculated from replicate to replicate (Fig. 3*C* and supplemental Fig. S6*A*–S6*D*). For the same bacteria across various biological replicates, the Pearson correlation factors were 0.894 in average. This good reproducibility again suggests a possible use of the method for bacterial quantification in urine.

### Transfer of the Signature on Different Instruments

**Fig. 4.**
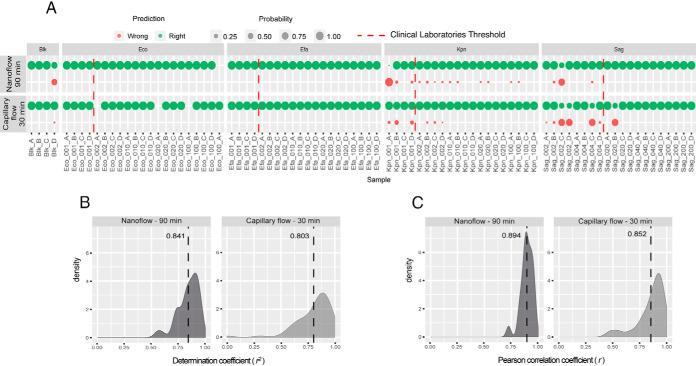
**Accuracy, linearity and reproducibility of the 'identification step' of the method performed in two experimental conditions: 90 min gradient at nanoflow rate with PRM acquisition on an Orbitrap Fusion instrument or 30 min gradient at capillary flow rate with PRM acquisition on a Q Exactive HF-X instrument.** *A*, Right (green) or wrong (red) prediction reported by the algorithm after peptidic signature monitoring by PRM associated to its probability, for five concentrations corresponding to five inoculation volumes (1, 2, 10, 20 and 100 μl or 2, 4, 20, 40 and 200 μl) of four bacteria (Eco, Efa, Kpn or Sag) in urine of four different healthy volunteers (A, B, C, and D), dotted red line corresponds to the commonly used clinical laboratories detection threshold of 1e5 CFU/ml; B, Distribution of the determination coefficients of the linearity curves obtained with the same samples across the five tested concentrations, the dotted line represents the average of all values; *C*, Distribution of the Pearson correlation coefficients obtained by comparison of two biological replicates with the same samples, the dotted line represents the average of all values.

To demonstrate that the initially designed signature using an Orbitrap Fusion instrument coupled to a nanoflow chromatography system is transferable to other instruments in others laboratories, we have analyzed the same inoculated urines of healthy volunteers (four different bacteria, five concentrations) in PRM mode on a Q-Exactive HF-X instrument coupled to capillary chromatography in PRM mode. Indeed, chromatography at higher flow rate (1 μl/min) improves the robustness of peptide separation and detection. To reduce the turnaround time between sample collection and bacterial identification as much as possible, the chromatographic gradient was also reduced between the Orbitrap Fusion and the Q Exactive HF-X from 90 to 30 min. As for the Orbitrap Fusion data, the data collected from the Q Exactive HF-X were analyzed using Skyline with the same validation criteria and the resulting list of detected peptides was used by the prediction algorithm (Fig. 4*A*, supplemental Fig. S7 and supplemental Table S9*B*). Thus, in 94% of the cases, the method allowed the correct prediction of the bacteria initially inoculated in the samples. Errors were found only for some of the two lowest concentrations points and in all cases but one, the sample was predicted as blank.

We could observe that wrong predictions happened more frequently on *S.agalactiae* inoculations. This can be explained by the fact that this bacterium has been inoculated at a slightly lower level than the others but also by the fact that only 4 peptides of the signature are used to predict this species. In all wrong predictions on *S.agalactiae* inoculates only 2 peptides among 4 met our criteria and the samples were predicted as blank. But when looking at the data above the clinical threshold of 1 × 10^5^ CFU/ml, the accuracy was significantly improved to reach 100%.

Linearities were also calculated by plotting the intensities of detected peptides across the five bacterial concentrations inoculated. The median of determination coefficients (R^2^) of all peptides was 0.803 (Fig. 4*B* and supplemental Fig. S8*A*–S8*D*). In terms of reproducibility, four biological replicates were analyzed on the Q Exactive HF-X. Scatter plots showed very good reproducibility because the Pearson correlation factors were 0.852 on average across the various biological replicates from the same bacterium at the same concentration (Fig. 4*C* and supplemental Fig. S9*A*–S9*D*). However, lower Pearson correlation coefficient values were obtained for some peptides of *Streptococcus agalactiae.* Again, this can be explained by the fact that this species has been inoculated at lower concentration than the others (supplemental Table S7).

Again, the good results in terms of linearity and reproducibility obtained on the Q-Exactive HF-X instrument suggest its potential use for quantification of bacteria in urine.

### Validation on Patient Samples and Comparison to MALDI-TOF MS

**Fig. 5.**
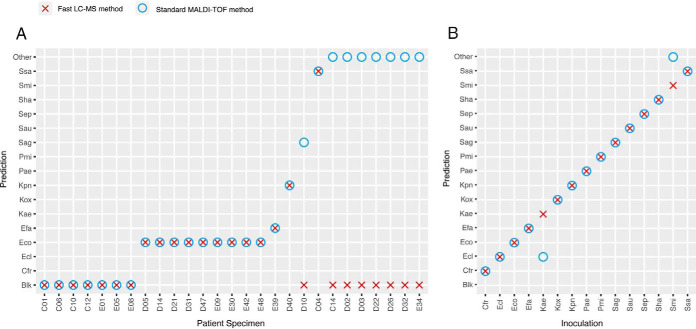
**Comparison of our fast LC-MS method and the standard MALDI-TOF method.** Prediction reported by the algorithm after peptidic signature monitoring by PRM without bacterial culture (red crosses) or by the MALDI Biotyper analysis after 24h hours bacterial culture (blue circles) on (*A*) 27 patients urine specimens or (*B*) 15 inoculations of bacterial species into urine from healthy volunteers.

To validate our method compared with conventional MALDI-TOF analysis, samples were collected from 27 patients. Aliquots of the samples were analyzed using either our pipeline without any culturing by monitoring the peptidic signature in PRM (nanoscale method) or with the standard MALDI-TOF method after 24 h bacterial culture and the predictions of both methods were compared (Fig. 5*A* and supplemental Tables S9*C* and S10). Most of the analyzed urines were determined to be not infected (*n* = 7) or infected by *E. coli* (*n* = 9) with both methods, whereas 4 other samples contained 4 different bacteria among our 15 targeted species. For those 20 patients we found a correlation between MALDI-TOF and LC-MS in 95% of cases. In seven other cases, the LC-MS/MS reported the urine as 'blank' (not infected) whereas the MALDI-TOF reported an identification marked as “probable” and limited at the genus level (*i.e.* without species mention). These results might be explained by a low level of contamination (below the level requiring anti-biotherapy) which prevented the LC-MS/MS method without culture to detect the signature peptides or by a contamination of the bacterial culture by non-pathogens, or infection of urines by species outside of our selection.

Among the 15 bacterial species detectable by our method, many of them have a quite low frequency in UTI and not found in urines tested. To validate the detection of these species with our method compared with MALDI-TOF, we inoculated an aliquot of healthy urine with each of the 15 bacteria at concentrations ranging from 3.32 × 10^5^ to 5.66 × 10^6^ CFU/ml (supplemental Table S7) and analyzed them with both pipelines. Our method found the correct inoculated bacterium in 100% of cases, whereas MALDI-TOF reported 2 errors (Fig. 5*B* and supplemental Tables S9*C* and S10). This lack of specificity in MALDI-TOF analysis might also explain some of the miscorrelations observed on patient samples.

## DISCUSSION

In this study, we developed a new strategy combining proteomics and machine learning for a fast, specific and accurate detection and identification of bacterial species present in urine without the need for time-consuming bacterial culture. We successfully applied our pipeline on the 15 bacterial species most commonly found in UTIs and obtained, in less than 4 h, high rates of prediction accuracy, especially when looking above the quantitative threshold commonly used by clinical laboratories to consider a urine as infected and requiring anti-biotherapy. These 15 species represent 84% of all UTIs, meaning that, by monitoring this peptidic signature, a wide majority of UTIs, as well as non-contaminated samples could be identified in less than four hours, allowing the possibility to delay the nonspecific antimicrobial treatment of the patients. Although the MALDI-TOF technology is able to discriminate thousands of species from pure colonies, our method could be improved in the future to allow the discrimination of more species causing UTIs. In that case, a new peptidic signature could be designed using the same pipeline where machine learning algorithms could be applied on newly acquired DIA data associated to the DIA data set available with this study.

Additionally, this proof of concept paves the way to the development of new peptidic signatures for the analysis of other types of clinical specimens (bronchoalveolar lavage (BAL), stool, hemoculture…) (53–55), but also for the detection of foodborne or waterborne pathogens (56, 57), to reduce the turnaround time required to obtain a genus- and/or species-specific identification of microorganisms by classical or molecular microbiology methods or MALDI-TOF mass spectrometry. In those cases, the sample preparation might need to be adapted for each type of specimen. However, centrifugation or microfiltration technics could be implemented to concentrate the bacteria and reduce the background proteins concentration. These strategies are widely used to isolate bacteria from water or in dairy industry for milk sterilization (58–60) and they have been applied on clinical samples to enrich for bacterial cells before detection (61–63). Sedimentation on spinning devices have also been reported to isolate bacterial from blood (64). For some of these applications, without any culture, the sensitivity of the method might be too low to detect the bacteria but, it is expected that a short-term culture in a liquid medium might be enough to reach a detectable level (<1 × 10^3^ CFU/ml for certain peptides) without the isolation of colonies on a culture plate. In all cases, the high specificity of the method, because of a fine selection of the signature peptides, leads to a great improvement to what can be obtained with other standard methods such as MALDI-TOF mass spectrometry or 16S rRNA sequencing. This would be particularly valuable for the epidemiological surveillance of specific pathogens, instead of relying on expensive and time-consuming whole genome sequencing (65, 66).

Moreover, the linearity and reproducibility of our method were evaluated and the obtained results suggest that the method could be used for quantification of bacterial cells in urine (for instance by addition of peptidic internal standards during the PRM monitoring). This would be particularly useful because, in the case of urine specimens, a real infection needs to be distinguished from low level bacterial contaminants and this could serve to prevent the inappropriate use of antibiotics (67). This quantification is currently done by a plate counting of the bacterial culture which is a long and inaccurate process. As reported here, once a signature is created, it can be transferred to other laboratories or other LC-MS systems working in parallel reaction monitoring (PRM). Because, only 1.52% of the peptide signals in our PRM data report a signal-to-noise ratio below 10, we can expect that the method could be transferable to selected reaction monitoring (SRM) mode on triple quadrupole (QQQ) instruments as some of those instruments have already been approved as Medical Devices for other applications (68) and are known for their low cost and robustness. However, in that case, it is expected to obtain lower signal-to-noise ratios because of the higher nonspecific background in QQQ instruments. The transitions to monitor should also be carefully selected for each peptide of the signature and a fine tuning of the source voltages and collision energy should be performed. But, once this optimization done, the method could be reused in routine on many samples. Moreover, it is also to note that the identification step could also been performed in DIA mode, because we have demonstrated on *E. coli* inoculates that, despite much more nonspecific interferences, prediction rate where similar for both PRM or DIA peptide signature monitoring (data not shown). This finding opens the possibility for monitoring larger signatures (> 100 peptides) even with short gradients. Although the turnaround time to identify bacterial contaminants with this method is short (<4 h), the non-parallelizable chromatographic time might limits its use in laboratories analyzing a high number of patient samples every day. Nevertheless, we showed that this time can be reduced from 90 to 30 min and could be even more shortened with a better acquisition scheduling, some optimization of the LC gradient or use of high-throughput LC devices (69). Sample preparation time could also be automated and optimized, for instance, the trypsin digestion here done in one hour might be reduced to a couple of minutes as reported in the literature (70, 71). Finally, in a context where the emergence of resistant bacterial strains poses a global public health threat (9, 72, 73), the development of fast methods for bacterial typing becomes essential. Indeed, broad spectrum antimicrobials are commonly prescribed to patients before obtaining the results of the clinical microbiological analysis, a practice that might not be sufficient to control the infection, especially when one considers the risk posed by antibiotic-resistant species and their transmission in the hospital environment or the community (74). For instance, *Staphylococcus aureus* has developed resistance to many antimicrobial drugs including last resort antibiotics and expresses an arsenal of virulence factors (75–77). Or, in agriculture, the systematic use of antibiotics in farming leads to the selection of resistant bacteria that have been found in commercial food products (78). The accessibility with proteomics methods of the proteins involved in the resistance or virulence processes might constitute a challenge. However, several studies already reported the use of MALDI-TOF and LC-MS/MS to detect changes in the proteome of sensitive *versus* resistance strains (79–81). Thus, by including specific peptides belonging to proteins involved in resistance or virulence mechanisms in our peptidic signature, we could provide a measure of the risk associated to resistance or virulence and provide additional microbial information a physician could use to prescribe an appropriate antibiotic to a patient, thereby reducing the use of broad-spectrum antibiotics.

Finally, we anticipate that the constant improvement in sensitivity, mass accuracy and acquisition speed of mass spectrometers will contribute in the future to improve the limit and precision of specific bacterial strains detection, making even more relevant the use of LC-MS/MS methods in microbiology.

## DATA AVAILABILITY

The raw mass spectrometry data are publicly available on ProteomeXchange (www.proteomexchange.org) on the following identifiers: PXD013885 (DDA data set), PXD013888 (DIA data set) and PXD014970 (PRM data set).

## Supplementary Material

Supp_Fig_I

Supp_Fig_II

Supp_Meth

Supp_Tab_1_7_8_10

Supp_Tab_2_part1

Supp_Tab_2_part2

Supp_Tab_3

Supp_Tab_4

Supp_Tab_6

SuppTable5

SuppTable9

## Footnotes

^1^ The abbreviations used are:

MALDI-TOF: Matrix Assisted Laser Desorption Ionization - Time Of Flight
DDA: Data Dependent Acquisition
DIA: Data Independent Acquisition
LC-MS/MS: Liquid Chromatography tandem Mass Spectrometry
PRM: Parallel Reaction Monitoring
SRM: Selected Reaction Monitoring
UTI: Urinary tract infection(s)
Cfr: *Citrobacter freundii*
Ecl: *Enterobacter cloacae*
Eco: *Escherichia coli*
Efa: *Enterococcus faecalis*
Kae: *Klebsiella aerogenes*
Kox: *Klebsiella oxytoca*
Kpn: *Klebsiella pneumoniae*
Pae: *Pseudomonas aeruginosa*
Pmi: *Proteus mirabilis*
Sag: *Streptococcus agalactiae*
Sau: *Staphylococcus aureus*
Sep: *Staphylococcus epidermidis*
Sha: *Staphylococcus haemolyticus*
Smi: *Streptococcus mitis*
Ssa: *Staphylococcus saprophyticus.*

## References

[1] G. B. Prasad, Mousumi Debnath, and Prakash S. Bisen. (2012). Microbes concepts and applications. Hoboken: John Wiley & Sons. Available from: http://onlinelibrary.wiley.com/book/10.1002/9781118311912

[2] Murray PR, Baron EJ. (2003) American Society for Microbiology. Manual of clinical microbiology, 8th ed., Washington, D.C.: ASM Press

[3] Sharma, S., Kaur, N., Malhotra, S., Madan, P., Ahmad, W., and Hans, C. (2016) Serotyping and antimicrobial susceptibility pattern of Escherichia coli isolates from urinary tract infections in pediatric population in a tertiary care hospital. J. Pathog. 2016, 2548517 27047691 10.1155/2016/2548517 PMC4800102

[4] Buehler, S. S., Madison, B., Snyder, S. R., Derzon, J. H., Cornish, N. E., Saubolle, M. A., et al (2016) Effectiveness of practices to increase timeliness of providing targeted therapy for inpatients with bloodstream infections: a laboratory medicine best practices systematic review and meta-analysis. Clin. Microbiol. Rev. 29, 59–103 26598385 10.1128/CMR.00053-14 PMC4771213

[5] Kollef, M. H. (2008) Broad-spectrum antimicrobials and the treatment of serious bacterial infections: getting it right up front. Clin. Infect. Dis. 47 Suppl 1, S3–S13 18713047 10.1086/590061

[6] Leekha, S., Terrell, C. L., and Edson, R. S. (2011) General principles of antimicrobial therapy. Mayo Clin. Proc. 86, 156–167 21282489 10.4065/mcp.2010.0639 PMC3031442

[7] Adamus-Bialek, W., Baraniak, A., Wawszczak, M., Gluszek, S., Gad, B., Wrobel, K., Bator, P., Majchrzak, M., and Parniewski, P. (2018) The genetic background of antibiotic resistance among clinical uropathogenic Escherichia coli strains. Mol. Biol. Rep. 45, 1055–1065 30008141 10.1007/s11033-018-4254-0 PMC6156760

[8] Davies, S. C., Fowler, T., Watson, J., Livermore, D. M., and Walker, D. (2013) Annual Report of the Chief Medical Officer: infection and the rise of antimicrobial resistance. Lancet 381, 1606–1609 23489756 10.1016/S0140-6736(13)60604-2

[9] WHO. (2014) Antimicrobial resistance: global report on surveillance. Geneva, switzerland: World Health Organization

[10] Woo, P. C., Lau, S. K., Teng, J. L., Tse, H., and Yuen, K. Y. (2008) Then and now: use of 16S rDNA gene sequencing for bacterial identification and discovery of novel bacteria in clinical microbiology laboratories. Clin. Microbiol. Infect. 14, 908–934 18828852 10.1111/j.1469-0691.2008.02070.x

[11] Balloux, F., Bronstad Brynildsrud, O., van Dorp, L., Shaw, L. P., Chen, H., Harris, K. A., Wang, H., and Eldholm, V. (2018) From theory to practice: translating whole-genome sequencing (WGS) into the clinic. Trends Microbiol. 26, 1035–1048 30193960 10.1016/j.tim.2018.08.004 PMC6249990

[12] Li, W., Raoult, D., and Fournier, P. E. (2009) Bacterial strain typing in the genomic era. FEMS Microbiol. Rev. 33, 892–916 19453749 10.1111/j.1574-6976.2009.00182.x

[13] Deurenberg, R. H., Bathoorn, E., Chlebowicz, M. A., Couto, N., Ferdous, M., Garcia-Cobos, S., Kooistra-Smid, A. M., Raangs, E. C., Rosema, S., Veloo, A. C., Zhou, K., Friedrich, A. W., and Rossen, J. W. (2017) Application of next generation sequencing in clinical microbiology and infection prevention. J. Biotechnol. 243, 16–24 28042011 10.1016/j.jbiotec.2016.12.022

[14] Field, D., Hughes, J., and Moxon, E. R. (2004) Using the genome to understand pathogenicity. Methods Mol. Biol. 266, 261–287 15148423 10.1385/1-59259-763-7:261

[15] Tagini, F., and Greub, G. (2017) Bacterial genome sequencing in clinical microbiology: a pathogen-oriented review. Eur. J. Clin. Microbiol. Infect. Dis. 36, 2007–2020 28639162 10.1007/s10096-017-3024-6 PMC5653721

[16] Fagerquist, C. K., Garbus, B. R., Miller, W. G., Williams, K. E., Yee, E., Bates, A. H., Boyle, S., Harden, L. A., Cooley, M. B., and Mandrell, R. E. (2010) Rapid identification of protein biomarkers of Escherichia coli O157:H7 by matrix-assisted laser desorption ionization-time-of-flight-time-of-flight mass spectrometry and top-down proteomics. Anal. Chem. 82, 2717–2725 20232878 10.1021/ac902455d

[17] Singhal, N., Kumar, M., Kanaujia, P. K., and Virdi, J. S. (2015) MALDI-TOF mass spectrometry: an emerging technology for microbial identification and diagnosis. Front. Microbiol. 6, 791 26300860 10.3389/fmicb.2015.00791 PMC4525378

[18] Angeletti, S. (2017) Matrix assisted laser desorption time of flight mass spectrometry (MALDI-TOF MS) in clinical microbiology. J. Microbiol. Methods 138, 20–29 27613479 10.1016/j.mimet.2016.09.003

[19] Sloan, A., Wang, G., and Cheng, K. (2017) Traditional approaches versus mass spectrometry in bacterial identification and typing. Clin. Chim. Acta 473, 180–185 28866114 10.1016/j.cca.2017.08.035

[20] Marko, D. C., Saffert, R. T., Cunningham, S. A., Hyman, J., Walsh, J., Arbefeville, S., Howard, W., Pruessner, J., Safwat, N., Cockerill, F. R., Bossler, A. D., Patel, R., and Richter, S. S. (2012) Evaluation of the Bruker Biotyper and Vitek MS matrix-assisted laser desorption ionization-time of flight mass spectrometry systems for identification of nonfermenting gram-negative bacilli isolated from cultures from cystic fibrosis patients. J. Clin. Microbiol. 50, 2034–2039 22495566 10.1128/JCM.00330-12 PMC3372130

[21] Cheng, K., Chui, H., Domish, L., Hernandez, D., and Wang, G. (2016) Recent development of mass spectrometry and proteomics applications in identification and typing of bacteria. Proteomics Clin. Appl. 10, 346–357 26751976 10.1002/prca.201500086 PMC5067657

[22] Clark, C. G., Kruczkiewicz, P., Guan, C., McCorrister, S. J., Chong, P., Wylie, J., van Caeseele, P., Tabor, H. A., Snarr, P., Gilmour, M. W., Taboada, E. N., and Westmacott, G. R. (2013) Evaluation of MALDI-TOF mass spectroscopy methods for determination of Escherichia coli pathotypes. J. Microbiol. Methods 94, 180–191 23816532 10.1016/j.mimet.2013.06.020

[23] Gekenidis, M. T., Studer, P., Wuthrich, S., Brunisholz, R., and Drissner, D. (2014) Beyond the matrix-assisted laser desorption ionization (MALDI) biotyping workflow: in search of microorganism-specific tryptic peptides enabling discrimination of subspecies. Appl. Environ. Microbiol. 80, 4234–4241 24795381 10.1128/AEM.00740-14 PMC4068689

[24] Ferreira, L., Sanchez-Juanes, F., Munoz-Bellido, J. L., and Gonzalez-Buitrago, J. M. (2011) Rapid method for direct identification of bacteria in urine and blood culture samples by matrix-assisted laser desorption ionization time-of-flight mass spectrometry: intact cell vs. extraction method. Clin. Microbiol. Infect 17, 1007–1012 20718803 10.1111/j.1469-0691.2010.03339.x

[25] Wuppenhorst, N., Consoir, C., Lorch, D., and Schneider, C. (2012) Direct identification of bacteria from charcoal-containing blood culture bottles using matrix-assisted laser desorption/ionisation time-of-flight mass spectrometry. Eur. J. Clin. Microbiol. Infect Dis. 31, 2843–2850 22639175 10.1007/s10096-012-1638-2

[26] Jeverica, S., Nagy, E., Mueller-Premru, M., and Papst, L. (2018) Sample preparation method influences direct identification of anaerobic bacteria from positive blood culture bottles using MALDI-TOF MS. Anaerobe. 54, 231–235 29861277 10.1016/j.anaerobe.2018.05.003

[27] Wang, H., Drake, S. K., Yong, C., Gucek, M., Lyes, M. A., Rosenberg, A. Z., Soderblom, E., Arthur Moseley, M., Dekker, J. P., and Suffredini, A. F. (2017) A genoproteomic approach to detect peptide markers of bacterial respiratory pathogens. Clin. Chem. 63, 1398–1408 28588123 10.1373/clinchem.2016.269647 PMC10863334

[28] Wang, H., Drake, S. K., Yong, C., Gucek, M., Tropea, M., Rosenberg, A. Z., Dekker, J. P., and Suffredini, A. F. (2016) A novel peptidomic approach to strain typing of clinical Acinetobacter baumannii isolates using mass spectrometry. Clin. Chem. 62, 866–875 27117471 10.1373/clinchem.2015.253468 PMC5548180

[29] Karlsson, R., Gonzales-Siles, L., Boulund, F., Svensson-Stadler, L., Skovbjerg, S., Karlsson, A., Davidson, M., Hulth, S., Kristiansson, E., and Moore, E. R. (2015) Proteotyping: Proteomic characterization, classification and identification of microorganisms–A prospectus. Syst. Appl. Microbiol. 38, 246–257 25933927 10.1016/j.syapm.2015.03.006

[30] Cheng, K., She, Y. M., Chui, H., Domish, L., Sloan, A., Hernandez, D., McCorrister, S., Ma, J., Xu, B., Reimer, A., Knox, J. D., and Wang, G. (2016) Mass spectrometry-based Escherichia coli H antigen/flagella typing: validation and comparison with traditional serotyping. Clin. Chem. 62, 839–847 27052506 10.1373/clinchem.2015.244236

[31] Jabbour, R. E., Deshpande, S. V., Stanford, M. F., Wick, C. H., Zulich, A. W., and Snyder, A. P. (2011) A protein processing filter method for bacterial identification by mass spectrometry-based proteomics. J. Proteome Res. 10, 907–912 21126090 10.1021/pr101086a

[32] Boulund, F., Karlsson, R., Gonzales-Siles, L., Johnning, A., Karami, N., Al-Bayati, O., Åhrén, C., Moore, E. R. B., and Kristiansson, E. (2017) Typing and characterization of bacteria using bottom-up tandem mass spectrometry proteomics. Mol. Cell Proteomics 16, 1052–1063 28420677 10.1074/mcp.M116.061721 PMC5461537

[33] Foxman, B. (2010) The epidemiology of urinary tract infection. Nat. Rev. Urol. 7, 653–660 21139641 10.1038/nrurol.2010.190

[34] Ronald A. (2002) The etiology of urinary tract infection: traditional and emerging pathogens. Am. J. Med. 113 Suppl 1A, 14S–19S 12113867 10.1016/s0002-9343(02)01055-0

[35] Rappsilber, J., Mann, M., and Ishihama, Y. (2007) Protocol for micro-purification, enrichment, pre-fractionation and storage of peptides for proteomics using StageTips. Nat. Protoc. 2, 1896–1906 17703201 10.1038/nprot.2007.261

[36] Kall, L., Canterbury, J. D., Weston, J., Noble, W. S., and MacCoss, M. J. (2007) Semi-supervised learning for peptide identification from shotgun proteomics data sets. Nat. Methods 4, 923–925 17952086 10.1038/nmeth1113

[37] Mesuere, B., Devreese, B., Debyser, G., Aerts, M., Vandamme, P., and Dawyndt, P. (2012) Unipept: tryptic peptide-based biodiversity analysis of metaproteome samples. J. Proteome Res. 11, 5773–5780 23153116 10.1021/pr300576s

[38] Mesuere, B., Van der Jeugt, F., Willems, T., Naessens, T., Devreese, B., Martens, L., and Dawyndt, P. (2018) High-throughput metaproteomics data analysis with Unipept: A tutorial. J. Proteomics 171, 11–22 28552653 10.1016/j.jprot.2017.05.022

[39] MacLean, B., Tomazela, D. M., Shulman, N., Chambers, M., Finney, G. L., Frewen, B., Kern, R., Tabb, D. L., Liebler, D. C., and MacCoss, M. J. (2010) Skyline: an open source document editor for creating and analyzing targeted proteomics experiments. Bioinformatics 26, 966–968 20147306 10.1093/bioinformatics/btq054 PMC2844992

[40] Egertson, J. D., MacLean, B., Johnson, R., Xuan, Y., and MacCoss, M. J. (2015) Multiplexed peptide analysis using data-independent acquisition and Skyline. Nat. Protoc. 10, 887–903 25996789 10.1038/nprot.2015.055 PMC5127711

[41] Reiter, L., Rinner, O., Picotti, P., Huttenhain, R., Beck, M., Brusniak, M. Y., Hengartner, M. O., and Aebersold, R. (2011) mProphet: automated data processing and statistical validation for large-scale SRM experiments. Nat. Methods 8, 430–435 21423193 10.1038/nmeth.1584

[42] Leclercq, M., Vittrant, B., Martin-Magniette, M. L., Scott Boyer, M. P., Perin, O., Bergeron, A., Fradet, Y., and Droit, A. (2019) Large-scale automatic feature selection for biomarker discovery in high-dimensional OMICs data. Front. Genet. 10, 452 31156708 10.3389/fgene.2019.00452 PMC6532608

[43] Frank, E., Hall, M. A., and Witten I. H. (2016) The WEKA Workbench. Online Appendix for “Data Mining: Practical Machine Learning Tools and Techniques”. In: Morgan Kaufmann [Internet]. Available from: www.cs.waikato.ac.nz/ml/weka/Witten_et_al_2016_appendix.pdf

[44] Gillet LC, Navarro P, Tate S, Rost H, Selevsek N, Reiter L, Bonner, R., and Aebersold, R. (2012) Targeted data extraction of the MS/MS spectra generated by data-independent acquisition: a new concept for consistent and accurate proteome analysis. Mol. Cell Proteomics 11, O111 016717 10.1074/mcp.O111.016717 PMC3433915 22261725

[45] Gillet, L. C., Leitner, A., and Aebersold, R. (2016) Mass spectrometry applied to bottom-up proteomics: entering the high-throughput era for hypothesis testing. Annu. Rev. Anal. Chem. 9, 449–472 10.1146/annurev-anchem-071015-041535 27049628

[46] Bruderer, R., Bernhardt, O. M., Gandhi, T., Xuan, Y., Sondermann, J., Schmidt, M., Gomez-Varela, D, and Reiter, L. (2017) Optimization of experimental parameters in data-independent mass spectrometry significantly increases depth and reproducibility of results. Mol. Cell Proteomics 16, 2296–2309 29070702 10.1074/mcp.RA117.000314 PMC5724188

[47] Camacho, D. M., Collins, K. M., Powers, R. K., Costello, J. C., and Collins, J. J. (2018) Next-generation machine learning for biological networks. Cell 173, 1581–1592 29887378 10.1016/j.cell.2018.05.015

[48] Matthews, B. W. (1975) Comparison of the predicted and observed secondary structure of T4 phage lysozyme. Biochim. Biophys. Acta 405, 442–451 1180967 10.1016/0005-2795(75)90109-9

[49] John, G. H., and Langley, P. (1995) Estimating continuous distributions in Bayesian classifiers. the Eleventh Conference on Uncertainty in Artificial Intelligence; Montreal, QC, Canada: Morgan Kaufmann Publishers Inc.

[50] Bouckaert, R. R. (2004) Bayesian Network Classifiers in Weka. New Zealand: Department of Computer Science, University of Waikato

[51] Hulten, G., Spencer, L., and Domingos, P. (2001) Mining time-changing data streams. seventh ACM SIGKDD international conference on knowledge discovery and data mining; San Francisco, CA: ACM

[52] Liebermeister, W., Noor, E., Flamholz, A., Davidi, D., Bernhardt, J., and Milo, R. (2014) Visual account of protein investment in cellular functions. Proc. Natl. Acad. Sci. U.S.A. 111, 8488–8493 24889604 10.1073/pnas.1314810111 PMC4060655

[53] Sung, J. Y., Hwang, Y., Shin, M. H., Park, M. S., Lee, S. H., Yong, D., and Lee, K. (2018) Utility of conventional culture and MALDI-TOF MS for identification of microbial communities in bronchoalveolar lavage fluid in comparison with the GS junior next generation sequencing system. Ann. Lab. Med. 38, 110–118 29214754 10.3343/alm.2018.38.2.110 PMC5736669

[54] He, Y., Li, H., Lu, X., Stratton, C. W., and Tang, Y. W. (2010) Mass spectrometry biotyper system identifies enteric bacterial pathogens directly from colonies grown on selective stool culture media. J. Clin. Microbiol. 48, 3888–3892 20844226 10.1128/JCM.01290-10 PMC3020868

[55] Haigh, J. D., Green, I. M., Ball, D., Eydmann, M., Millar, M., and Wilks, M. (2013) Rapid identification of bacteria from bioMerieux BacT/ALERT blood culture bottles by MALDI-TOF MS. Br. J. Biomed Sci. 70, 149–155 24400426 10.1080/09674845.2013.11669949

[56] Elbehiry, A., Marzouk, E., Hamada, M., Al-Dubaib, M., Alyamani, E., Moussa, I. M., AlRowaidhan, A., and Hemeg, HA. (2017) Application of MALDI-TOF MS fingerprinting as a quick tool for identification and clustering of foodborne pathogens isolated from food products. New Microbiol. 40, 269–278 28825446

[57] Dilger, T., Melzl, H., and Gessner, A. (2016) Rapid and reliable identification of waterborne Legionella species by MALDI-TOF mass spectrometry. J. Microbiol. Methods 127, 154–159 27260989 10.1016/j.mimet.2016.05.028

[58] Totaro, M., Valentini, P., Casini, B., Miccoli, M., Costa, A. L., and Baggiani, A. (2017) Experimental comparison of point-of-use filters for drinking water ultrafiltration. J. Hosp. Infect. 96, 172–176 28073586 10.1016/j.jhin.2016.11.017

[59] Fernandez Garcia, L., Alvarez Blanco, S., and Riera Rodriguez, F. A. (2013) Microfiltration applied to dairy streams: removal of bacteria. J. Sci. Food Agric. 93, 187–196 23169488 10.1002/jsfa.5935

[60] Brewster, J. D., and Paul, M. (2016) Short communication: Improved method for centrifugal recovery of bacteria from raw milk applied to sensitive real-time quantitative PCR detection of Salmonella spp. J. Dairy Sci. 99, 3375–3379 26971150 10.3168/jds.2015-9655

[61] Bernhardt, M., Pennell, D. R., Almer, L. S., and Schell, R. F. (1991) Detection of bacteria in blood by centrifugation and filtration. J. Clin. Microbiol. 29, 422–425 2037658 10.1128/jcm.29.3.422-425.1991 PMC269792

[62] Wilson, G., and Aitchison, L. B. (2007) The use of a combined enrichment-filtration technique for the isolation of Campylobacter spp. from clinical samples. Clin. Microbiol. Infect. 13, 643–644 17371536 10.1111/j.1469-0691.2007.01712.x

[63] Fourie, J., Loskutoff, N., and Huyser, C. (2012) Elimination of bacteria from human semen during sperm preparation using density gradient centrifugation with a novel tube insert. Andrologia. 44 Suppl 1, 513–517 21950521 10.1111/j.1439-0272.2011.01217.x

[64] Buchanan, C. M., Wood, R. L., Hoj, T. R., Alizadeh, M., Bledsoe, C. G., Wood, M. E., McClellan, D. S., Blanco, R., Hickey, C. L., Ravsten, T. V., Husseini, G. A., Robison, R. A., and Pitt, W. G. (2017) Rapid separation of very low concentrations of bacteria from blood. J. Microbiol. Methods 139, 48–53 28495585 10.1016/j.mimet.2017.05.004 PMC5533616

[65] Truong, D. T., Tett, A., Pasolli, E., Huttenhower, C., and Segata, N. (2017) Microbial strain-level population structure and genetic diversity from metagenomes. Genome Res. 27, 626–638 28167665 10.1101/gr.216242.116 PMC5378180

[66] Scholz, M., Ward, D. V., Pasolli, E., Tolio, T., Zolfo, M., Asnicar, F., Truong, D. T., Tett, A., Morrow, A. L., and Segata, N. (2016) Strain-level microbial epidemiology and population genomics from shotgun metagenomics. Nat. Methods 13, 435–438 26999001 10.1038/nmeth.3802

[67] Kwon, J. H., Fausone, M. K., Du, H., Robicsek, A., and Peterson, L. R. (2012) Impact of laboratory-reported urine culture colony counts on the diagnosis and treatment of urinary tract infection for hospitalized patients. Am. J. Clin. Pathol. 137, 778–784 22523217 10.1309/AJCP4KVGQZEG1YDM

[68] Heaney, L. M., Jones, D. J., and Suzuki, T. (2017) Mass spectrometry in medicine: a technology for the future? Future Sci. OA. 3, FSO213 28884010 10.4155/fsoa-2017-0053 PMC5583653

[69] Bache, N., Geyer, P. E., Bekker-Jensen, D. B., Hoerning, O., Falkenby, L., Treit, P. V., Doll, S., Paron, I., Müller, J. B., Meier, F., Olsen, J. V., Vorm, O., and Mann, M. (2018) A novel LC system embeds analytes in pre-formed gradients for rapid, ultra-robust proteomics. Mol. Cell Proteomics 17, 2284–2296 30104208 10.1074/mcp.TIR118.000853 PMC6210218

[70] Lesur, A., Varesio, E., and Hopfgartner, G. (2010) Accelerated tryptic digestion for the analysis of biopharmaceutical monoclonal antibodies in plasma by liquid chromatography with tandem mass spectrometric detection. J. Chromatogr. A. 1217, 57–64 19939394 10.1016/j.chroma.2009.11.011

[71] Kim, H., Kim, H. S., Lee, D., Shin, D., Shin, D., Kim, J., and Kim, J. (2017) Microwave-assisted protein digestion in a plate well for facile sampling and rapid digestion. Anal. Chem. 89, 10655–10660 28945068 10.1021/acs.analchem.7b02169

[72] WHO. (2012) The evolving threat of antimicrobial resistance: options for action. Geneva: World Health Organization

[73] Allcock, S., Young, E. H., Holmes, M., Gurdasani, D., Dougan, G., Sandhu, M. S., Solomon, L., and Török, M. E. (2017) Antimicrobial resistance in human populations: challenges and opportunities. Glob. Health Epidemiol. Genom. 2, e4 29276617 10.1017/gheg.2017.4 PMC5732576

[74] Laxminarayan, R., Matsoso, P., Pant, S., Brower, C., Rottingen, J. A., Klugman, K., and Davies, S. (2016) Access to effective antimicrobials: a worldwide challenge. Lancet 387, 168–175 26603918 10.1016/S0140-6736(15)00474-2

[75] Shorr, A. F. (2007) Epidemiology of staphylococcal resistance. Clin. Infect Dis. 45 Suppl 3, S171–S176 17712743 10.1086/519473

[76] Lakhundi, S., and Zhang, K. (2018) Methicillin-resistant Staphylococcus aureus: molecular characterization, evolution, and epidemiology. Clin. Microbiol. Rev. 31, pii: e00020–18 30209034 10.1128/CMR.00020-18 PMC6148192

[77] Oliveira, D., Borges, A., and Simoes, M. (2018) Staphylococcus aureus toxins and their molecular activity in infectious diseases. Toxins 10, E252 10.3390/toxins10060252 PMC6024779 29921792

[78] Landers, T. F., Cohen, B., Wittum, T. E., and Larson, E. L. (2012) A review of antibiotic use in food animals: perspective, policy, and potential. Public Health Rep. 127, 4–22 22298919 10.1177/003335491212700103 PMC3234384

[79] Park, A. J., Krieger, J. R., and Khursigara, C. M. (2016) Survival proteomes: the emerging proteotype of antimicrobial resistance. FEMS Microbiol. Rev. 40, 323–342 26790948 10.1093/femsre/fuv051

[80] Mekonnen, S. A., Palma Medina, L. M., Michalik, S., Loreti, M. G., Gesell Salazar, M., van Dijl, J. M., and Volker, U. (2019) Metabolic niche adaptation of community- and hospital-associated methicillin-resistant Staphylococcus aureus. J. Proteomics 193, 154–161 30321607 10.1016/j.jprot.2018.10.005

[81] Lin, M. H., Potel, C. M., Tehrani, K., Heck, A. J. R., Martin, N. I., and Lemeer, S. (2018) A new tool to reveal bacterial signaling mechanisms in antibiotic treatment and resistance. Mol. Cell Proteomics 17, 2496–2507 30232125 10.1074/mcp.RA118.000880 PMC6283303

