# Supplementary material for: Fast and Accurate Bacterial Species Identification in Urine Specimens Using LC-MS/MS Mass Spectrometry and Machine Learning
Source: Mol Cell Proteomics. 2019 Oct 4;18(12):2492–505. doi: 10.1074/mcp.TIR119.001559 (PMC6885708; doi:10.1074/mcp.TIR119.001559)

Supplementary Figure 1. **Statistics of Urine Specimen analyses at the microbiology laboratory of Enfant-Jesus Hospital in Quebec City, Canada.** The pie chart represents, for 8631 infected specimens, the proportion of each bacterial species identified by MALDI-TOF analysis using the standard procedure described in the Methods section over four months (January to April 2017).

Supplementary Figure 2. **Sequence Redundancy between the 15 bacterial species of interest.** (a) Heatmap represented the list of 31893 peptides sequences selected after DDA analysis of each pure species. Each sequence found in a species is indicated in dark blue. Hierarchical clustering was performed in rows and columns. (b) Using the same list of peptide sequences, the graph indicates how many peptides are specific to only one species when comparing 2 or more species (in frequency order in UTIs). (c) The bar plot represents the repartition of specific peptides when comparing 15 bacterial species.

Supplementary Figure 3. **Venn diagram of the peptide signatures given by machine learning classifiers.** (a) overlap of features found the 3 models used : Hoeffding tree, NaiveBayes and BayesNet; (b) overlap of features between the 3 models and the final 82 peptide signature

Supplementary Figure 4. **Heatmap of the peptidic signature corresponding to the 15 most frequently found bacteria in UTI.** Intensity of each of the 82 peptides identified by the Machine Learning algorithm is represented for the all low-level and high-level concentration replicates of urine inoculation for each bacteria of interest. Data are presented with a hierarchical clustering in rows and columns.

Supplementary Figure 5. **Linearity of the 'identification step' of the method performed with a 90 minutes gradient on an Orbitrap Fusion instrument.** Linearity curves were plotted for five concentrations corresponding to five inoculation volumes (1, 2, 10, 20 and 100μL or 2, 4, 20, 40 and 200μL) in urine of four different healthy volunteers (A, B, C, and D) for peptides of four bacteria: (a) *Escherichia coli*, (b) *Enterococcus faecalis*, (c) *Klebsiella pneumoniae*, (d) *Streptococcus agalactiae*. Dotted red line corresponds to the commonly used clinical laboratories detection threshold of 1e5 CFU/mL.

Supplementary Figure 6. **Reproducibility the 'identification step' of the method performed with a 90 minutes gradient on an Orbitrap Fusion instrument.** Scatter plots and Pearson correlation coefficient are presented for four biological replicates corresponding to all inoculation volumes in urine of four different healthy volunteers (A, B, C, and D) for four bacteria: (a) *Escherichia coli*, (b) *Enterococcus faecalis*, (c) *Klebsiella pneumoniae*, (d) *Streptococcus agalactiae*.

Supplementary Figure 7. **Accuracy of the 'identification step' of the method performed with a 30 minutes gradient on a Q-Exactive HF-X instrument.** Predictions reported by the algorithm after peptidic signature monitoring by PRM associated with its probability (light blue : high probability, dark blue : low probability) for five concentrations corresponding

to five inoculation volumes (1, 2, 10, 20 and 100µL or 2, 4, 20, 40 and 200µL) of four bacteria (Eco, Efa, Kpn or Sag) in urine of four different healthy volunteers (A, B, C, and D), dotted red line corresponds to the commonly used clinical laboratories detection threshold of 1e5 CFU/mL.

Supplementary Figure 8. **Linearity of the 'identification step' of the method performed with a 30 minutes gradient on a Q-Exactive HF-X instrument.** Linearity curves were plotted for five concentrations corresponding to five inoculation volumes (1, 2, 10, 20 and 100µL or 2, 4, 20, 40 and 200µL) in urine of four different healthy volunteers (A, B, C, and D) for peptides of four bacteria: (a) *Escherichia coli*, (b) *Enterococcus faecalis*, (c) *Klebsiella pneumoniae*, (d) *Streptococcus agalactiae*. Dotted red line corresponds to the commonly used clinical laboratories detection threshold of 1e5 CFU/mL.

Supplementary Figure 9. **Reproducibility the 'identification step' of the method performed with a 30 minutes gradient on a Q-Exactive HF-X instrument.** Scatter plots and Pearson correlation coefficient are presented for four biological replicates corresponding to all inoculation volumes in urine of four different healthy volunteers (A, B, C, and D) for four bacteria: (a) *Escherichia coli*, (b) *Enterococcus faecalis*, (c) *Klebsiella pneumoniae*, (d) *Streptococcus agalactiae*.

Supplementary figure 1

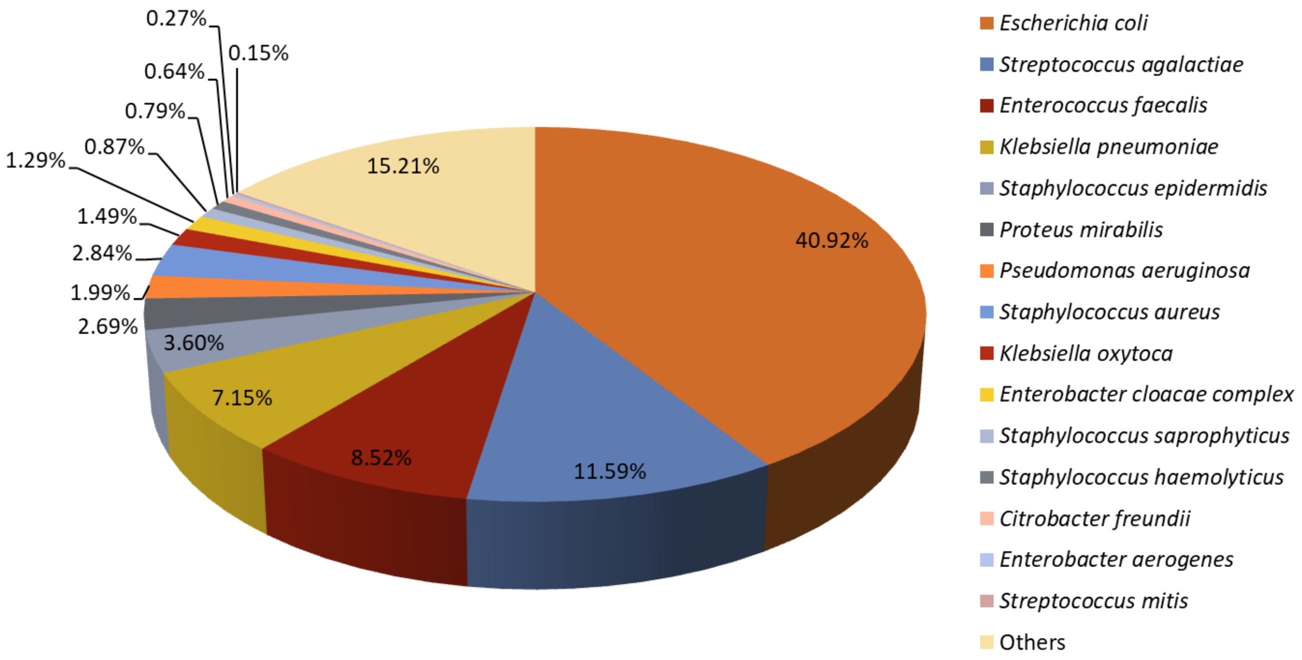

Supplementary Figure 2

a

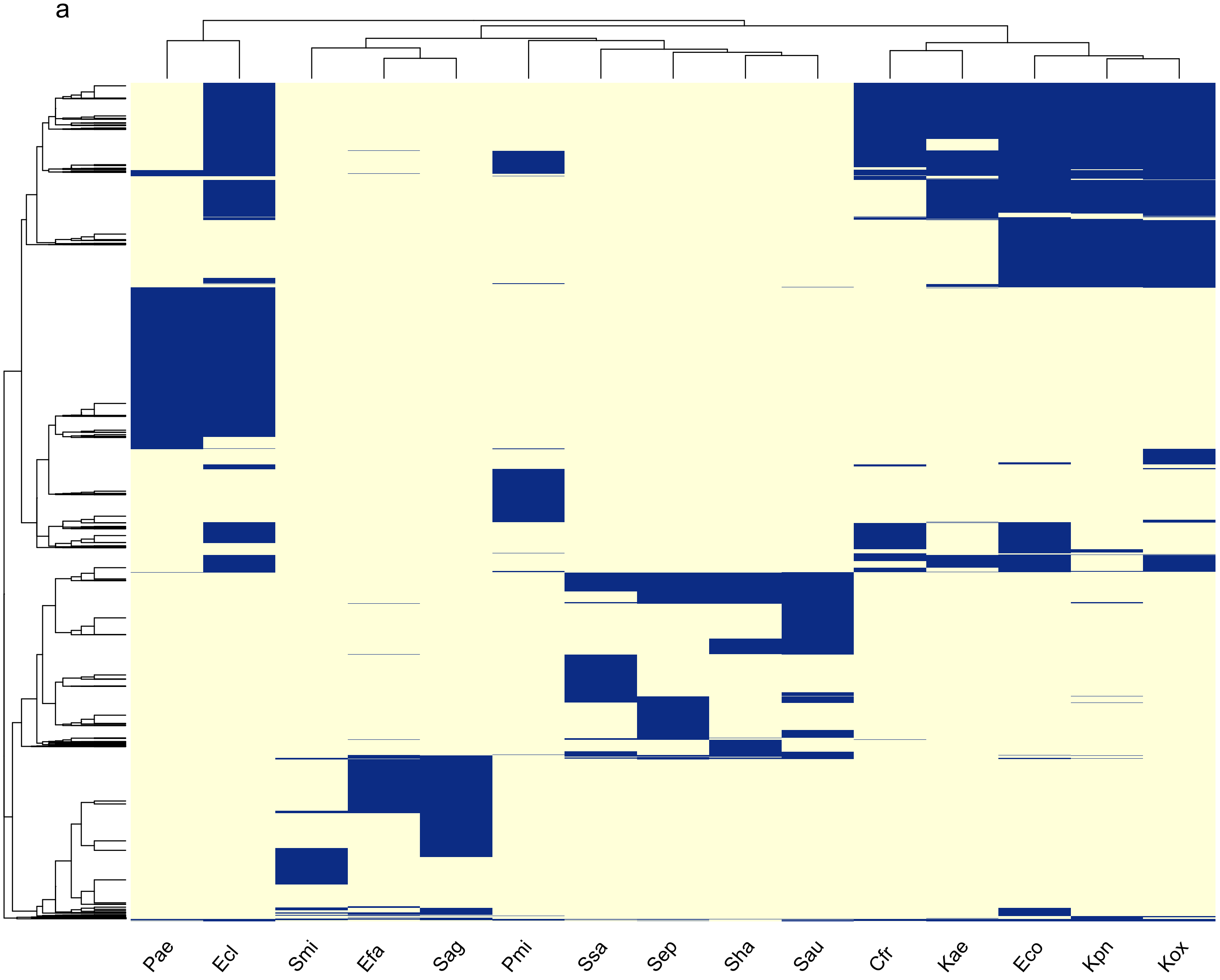

b

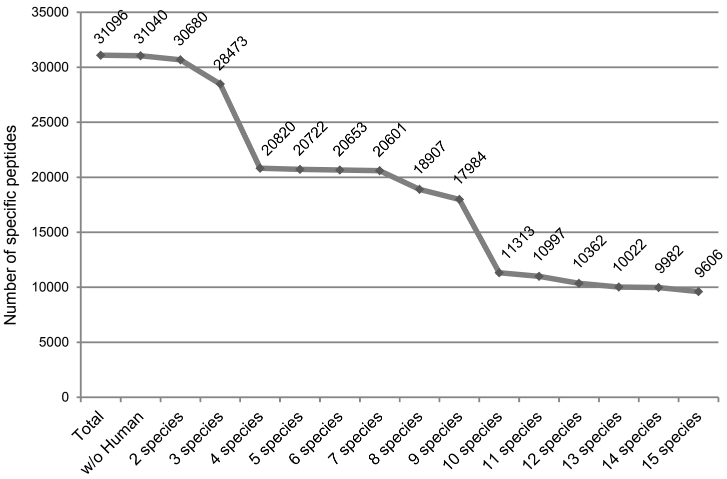

c

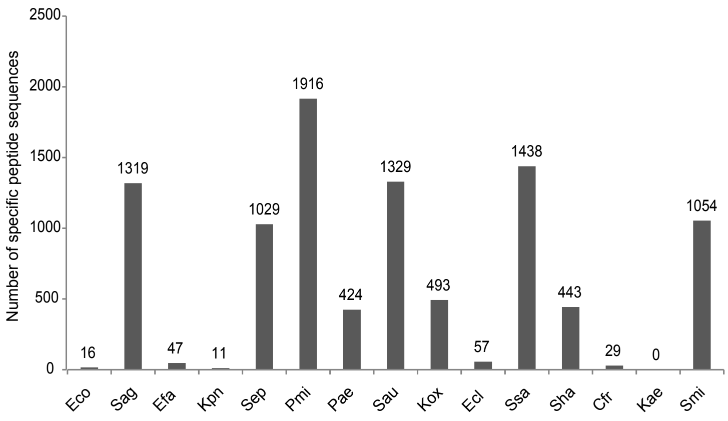

Supplementary figure 3

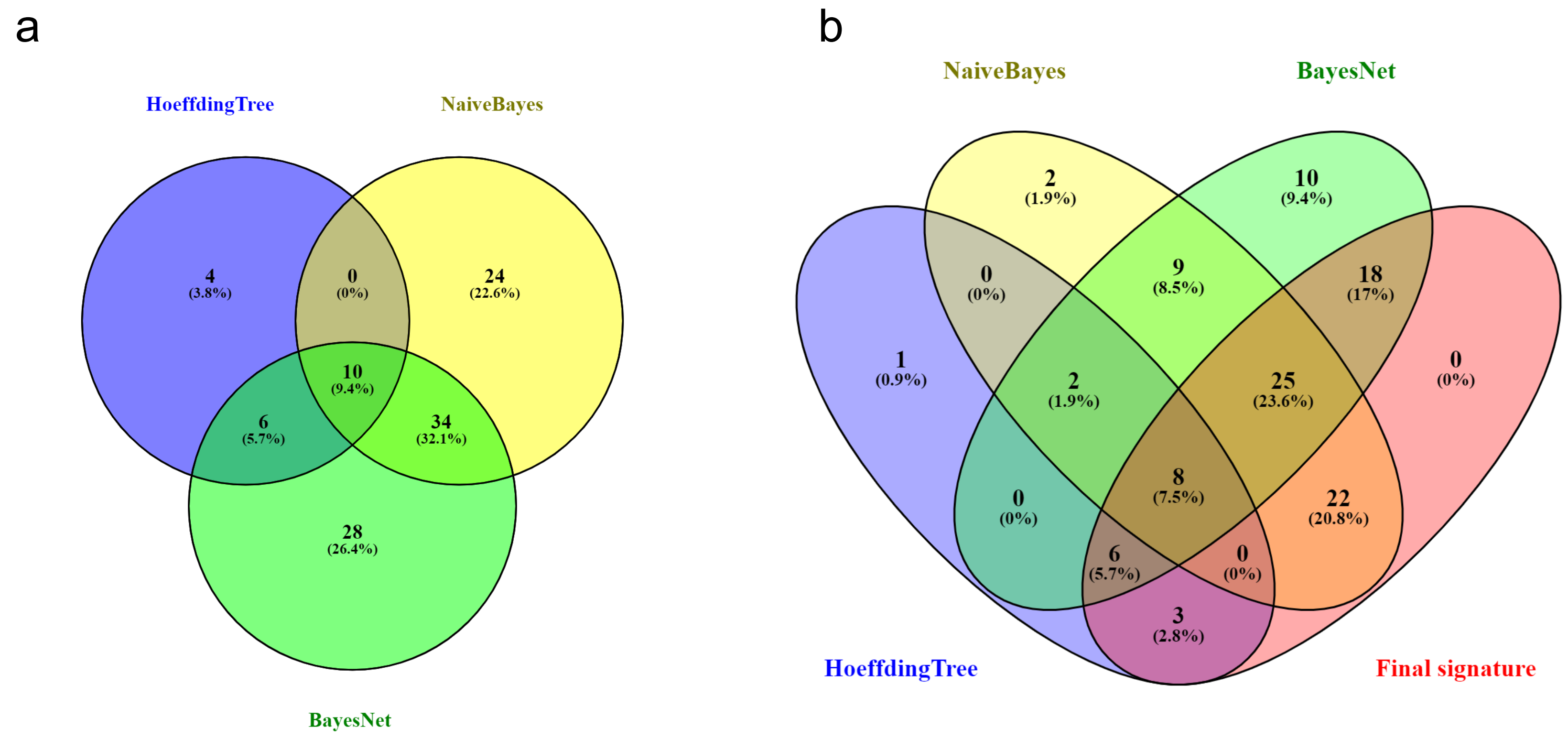

Supplementary Figure 4

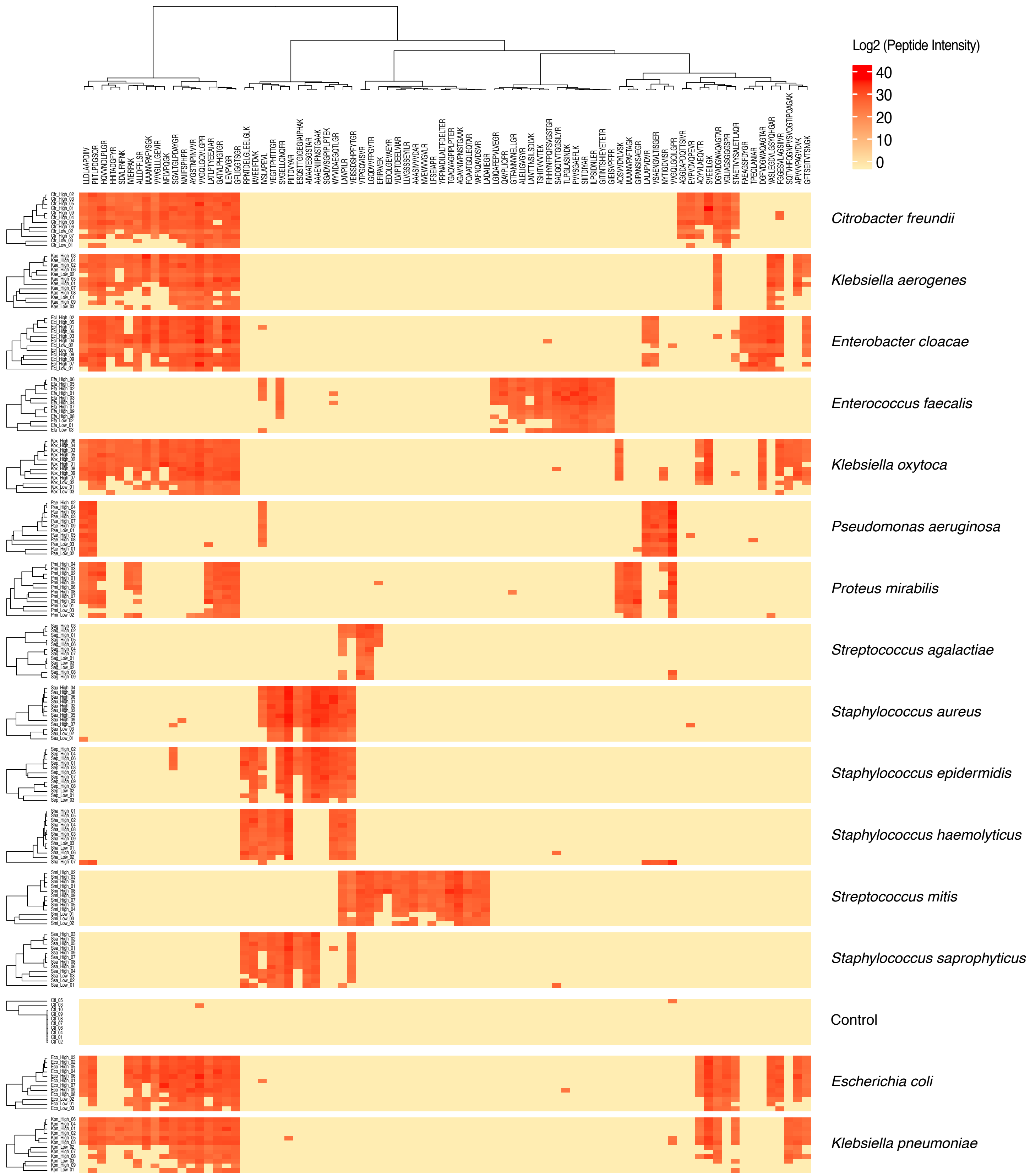

Supplementary Figure 5

a

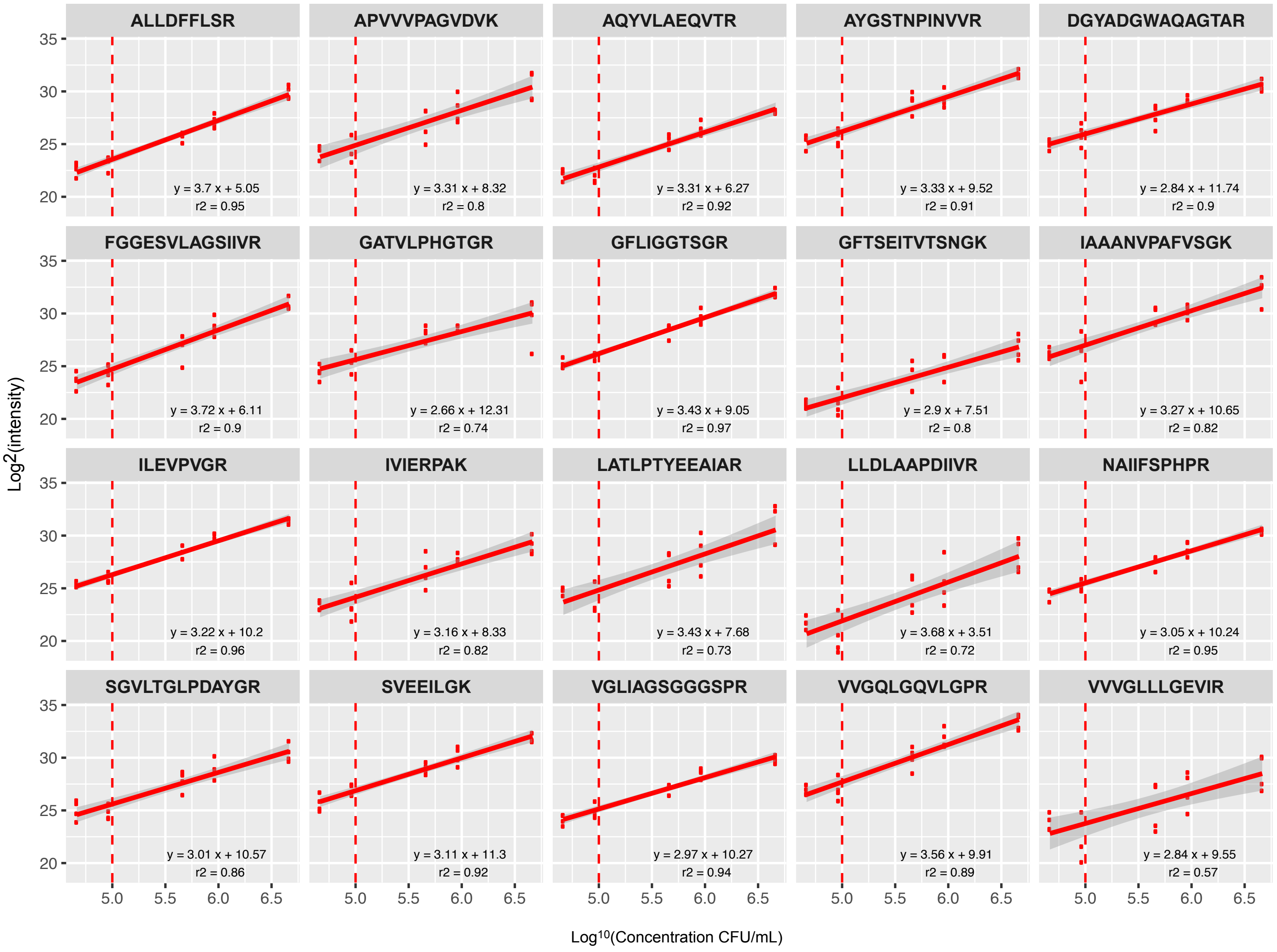

Supplementary Figure 5

b

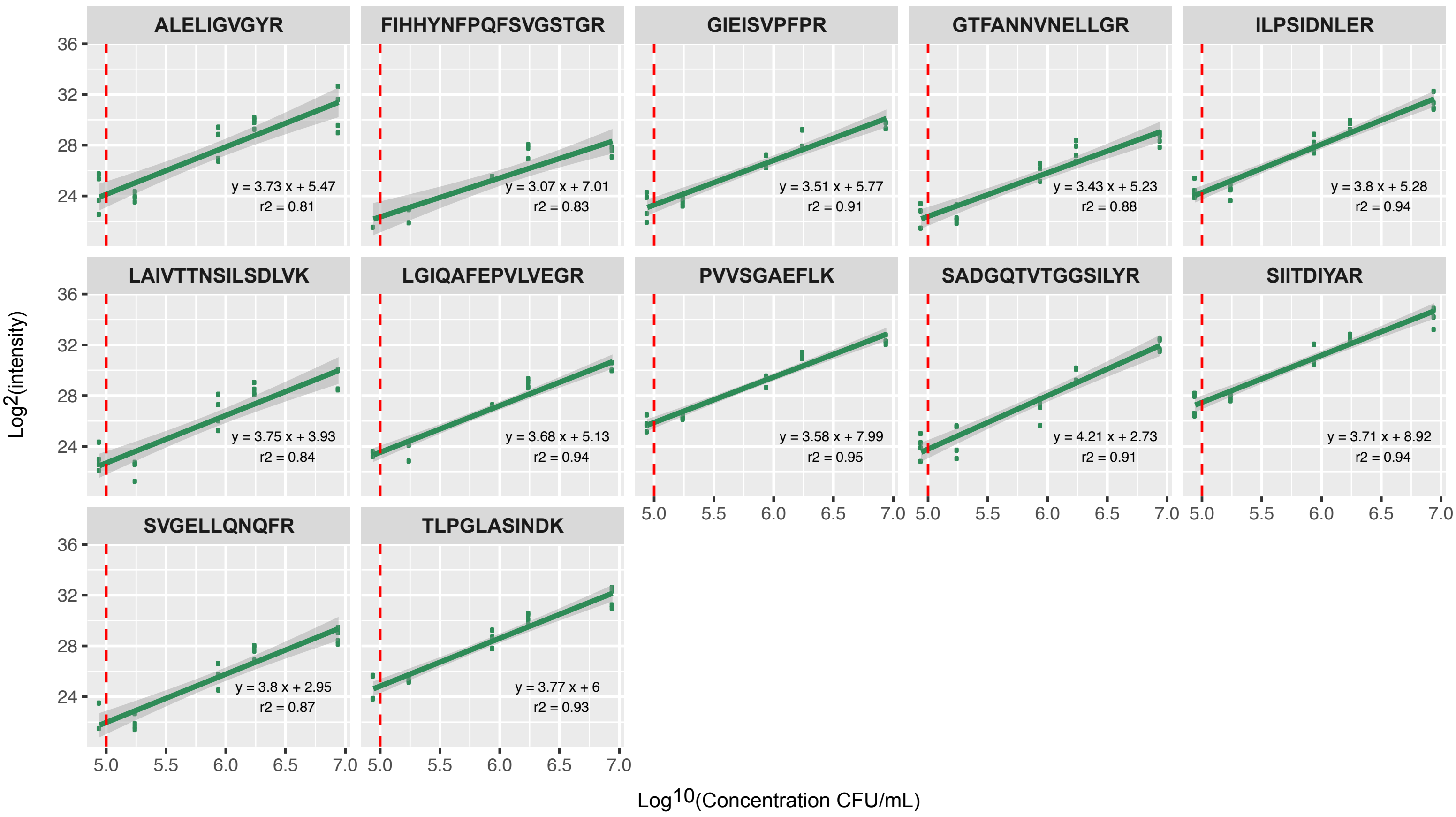

Supplementary Figure 5

c

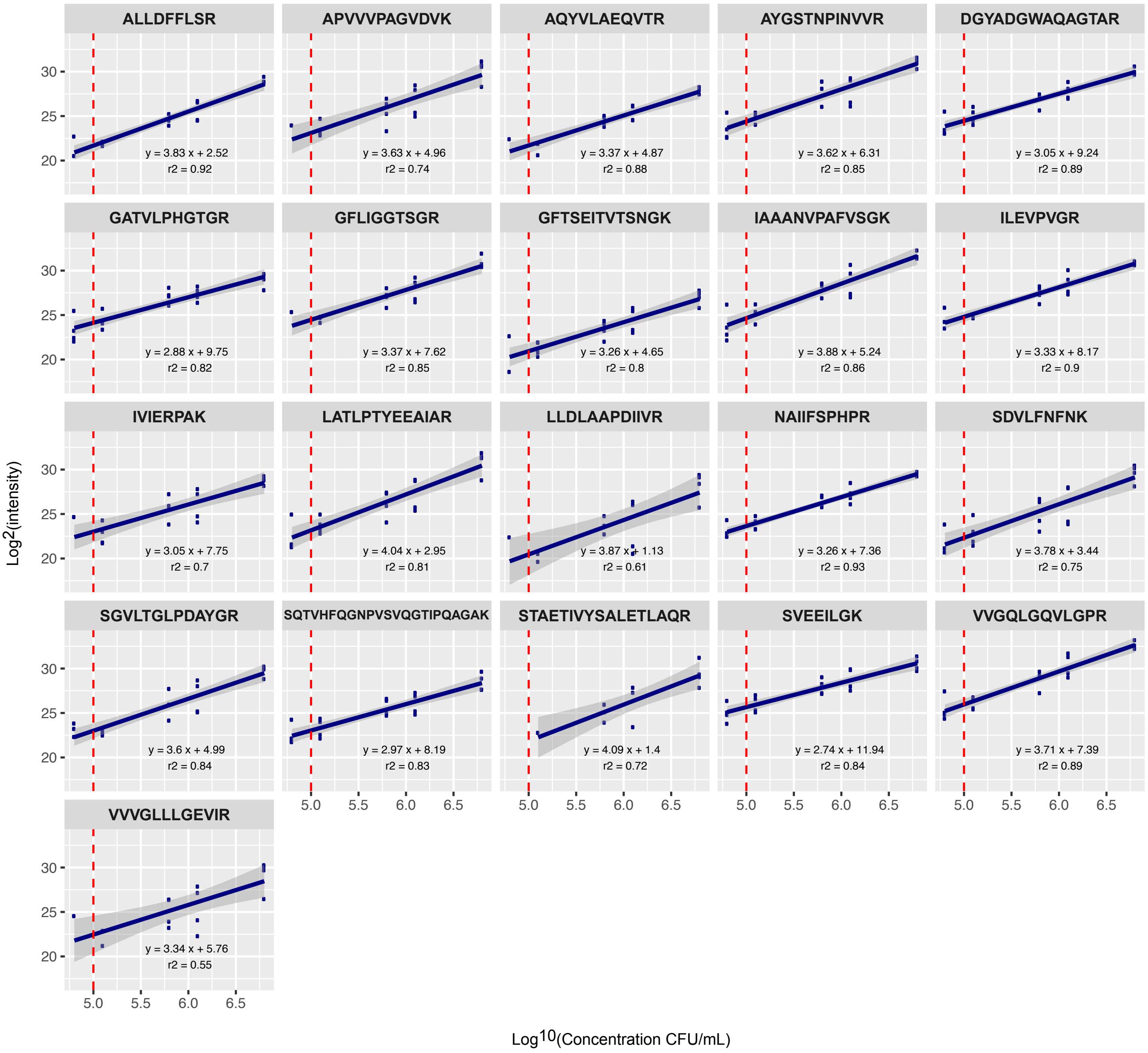

Supplementary Figure 5

d

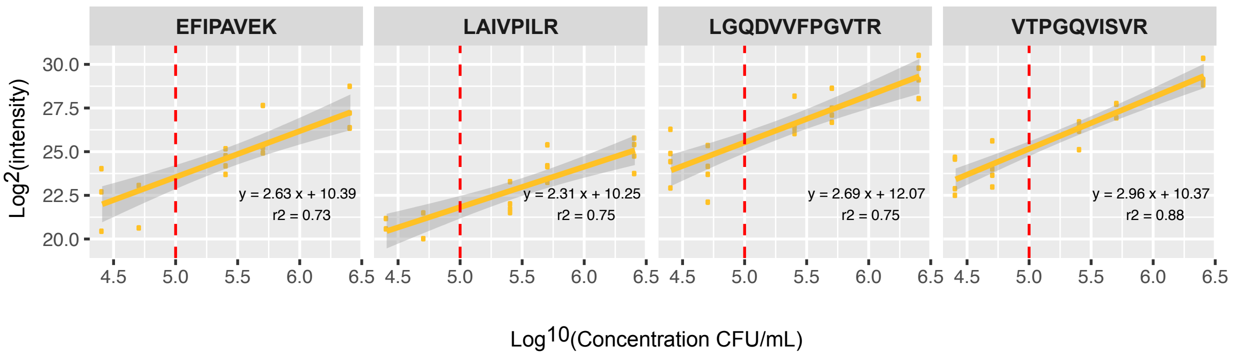

Supplement: Supp_Fig_I [file 152789_2_supp_402450_py28d4.pdf]
