# Supplementary material for: Fast and Accurate Bacterial Species Identification in Urine Specimens Using LC-MS/MS Mass Spectrometry and Machine Learning
Source: Mol Cell Proteomics. 2019 Oct 4;18(12):2492–505. doi: 10.1074/mcp.TIR119.001559 (PMC6885708; doi:10.1074/mcp.TIR119.001559)

Supplementary Figure 6

a

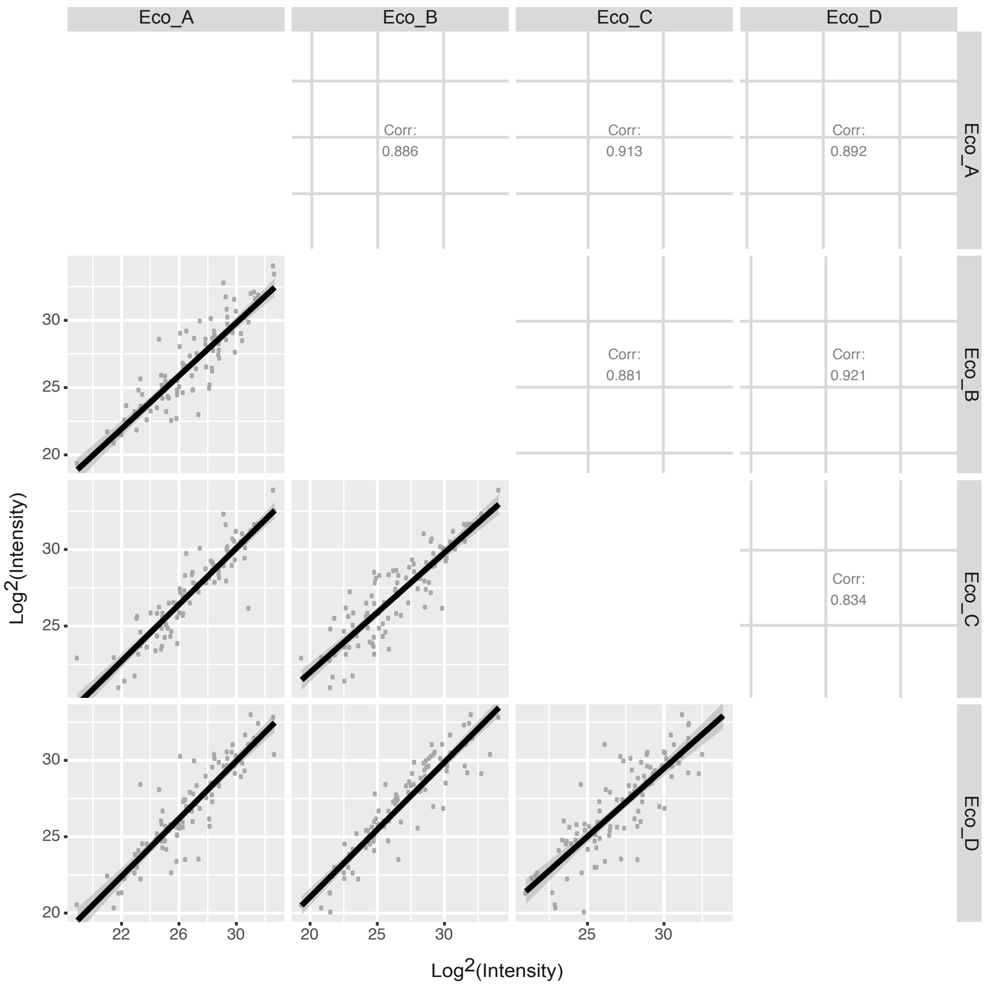

b

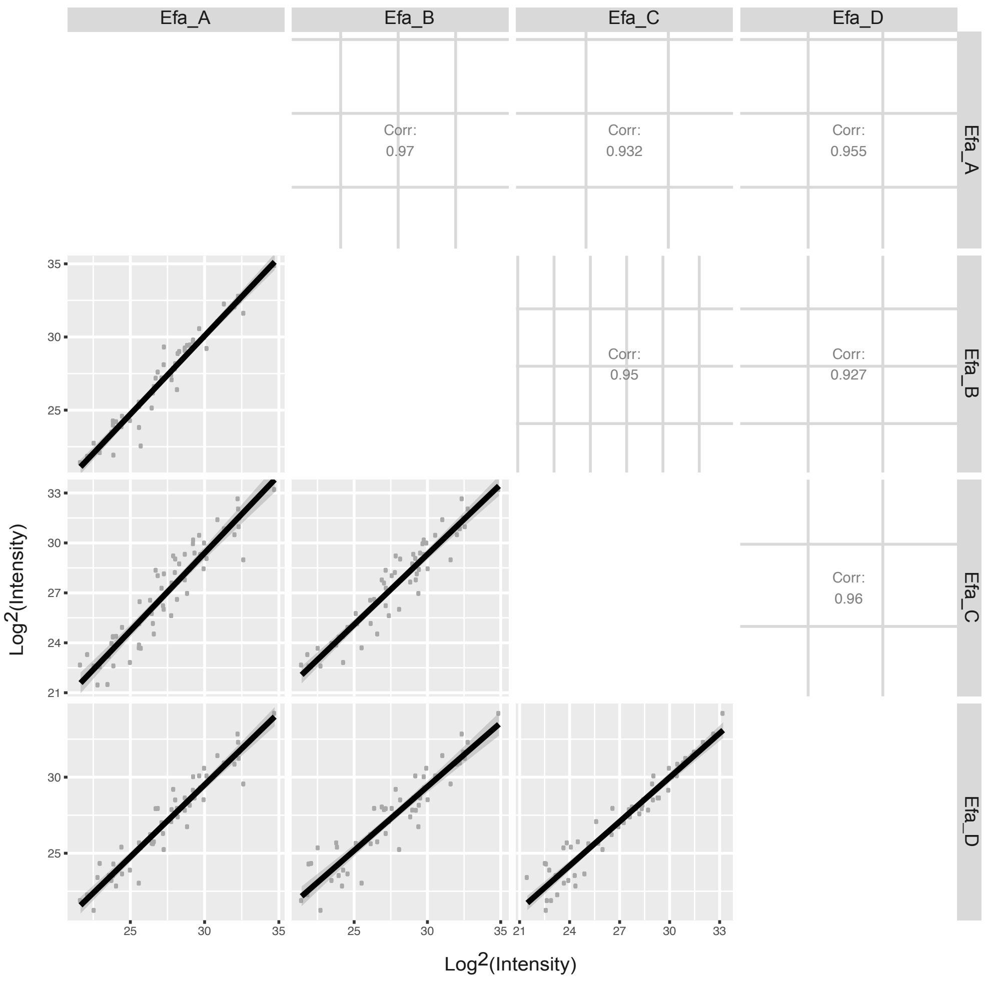

Supplementary Figure 6

c

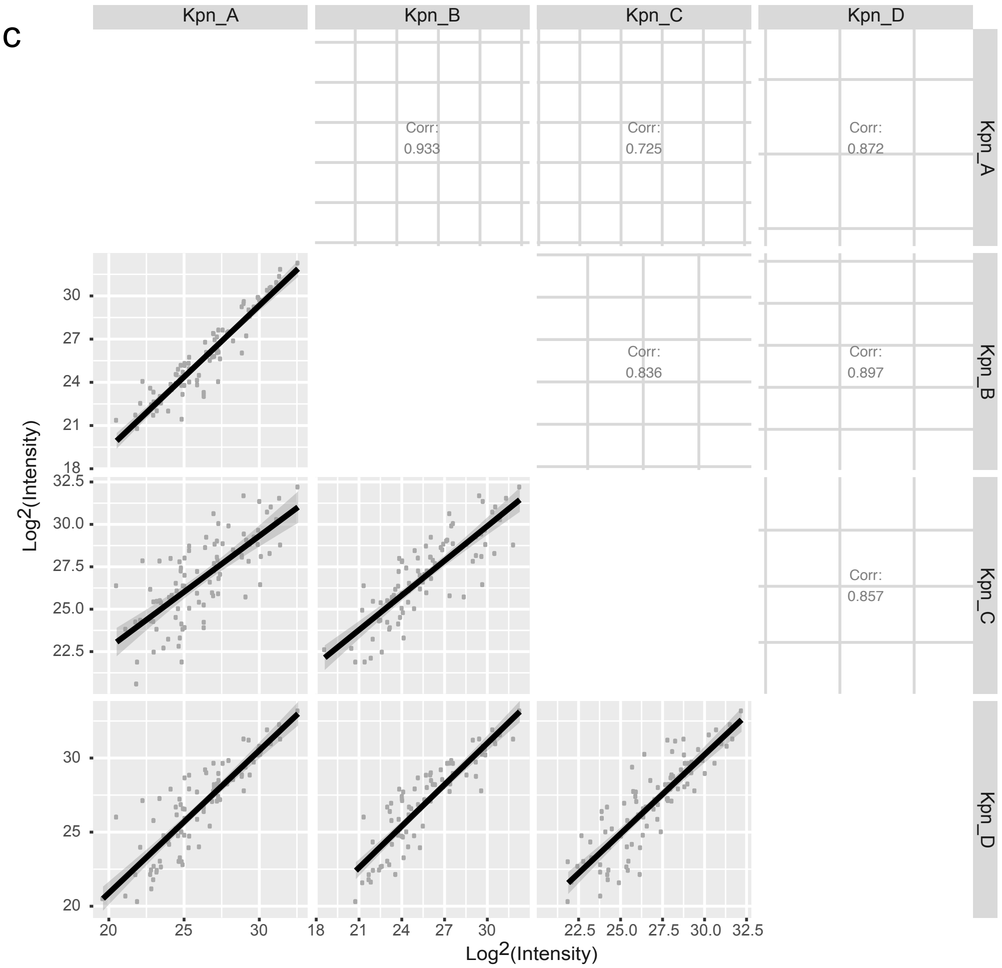

d

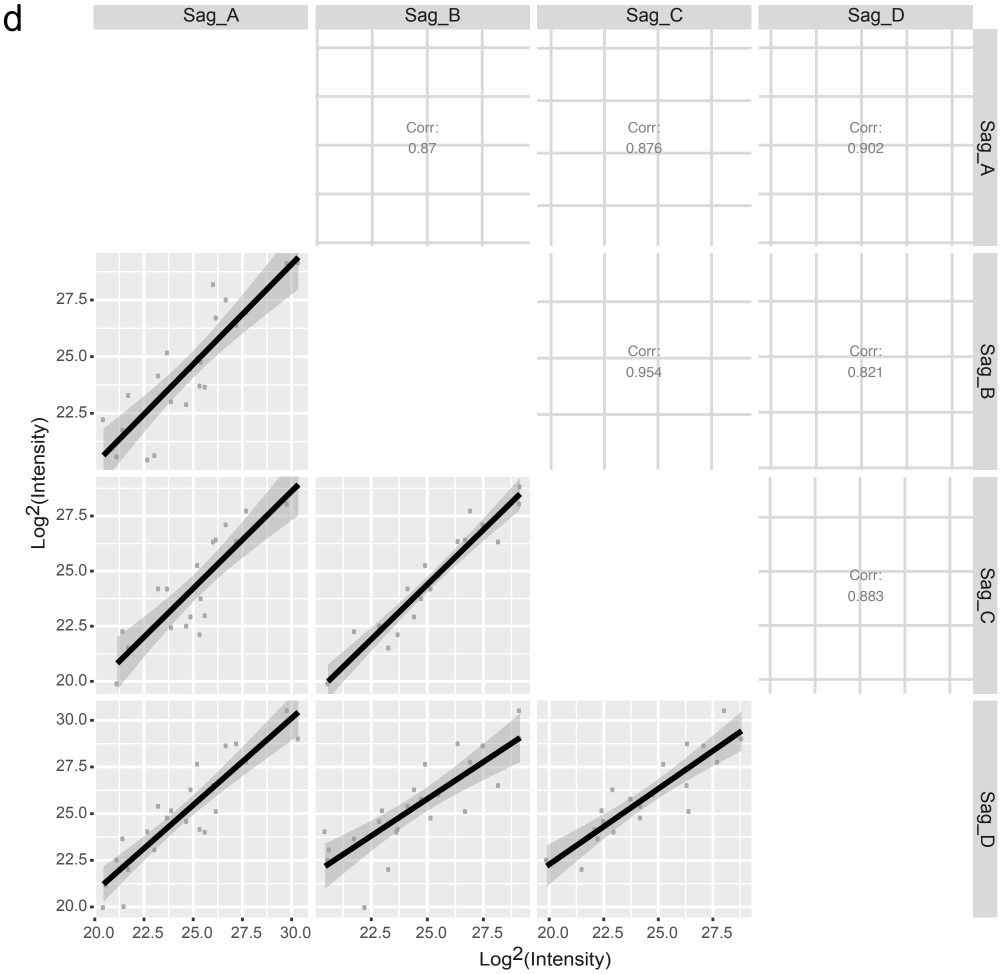

Supplementary Figure 7

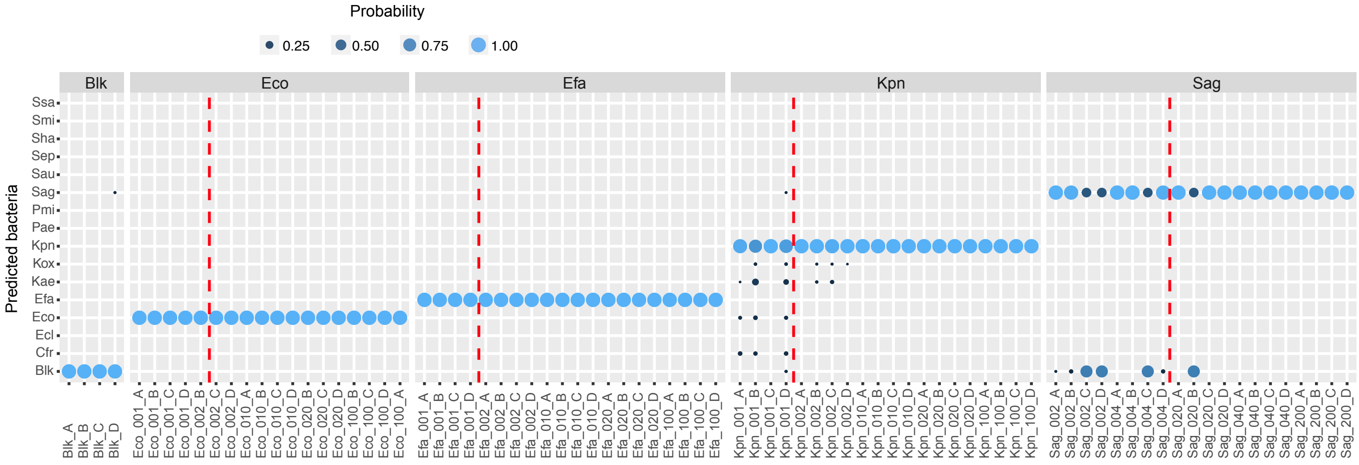

Supplementary Figure 8

a

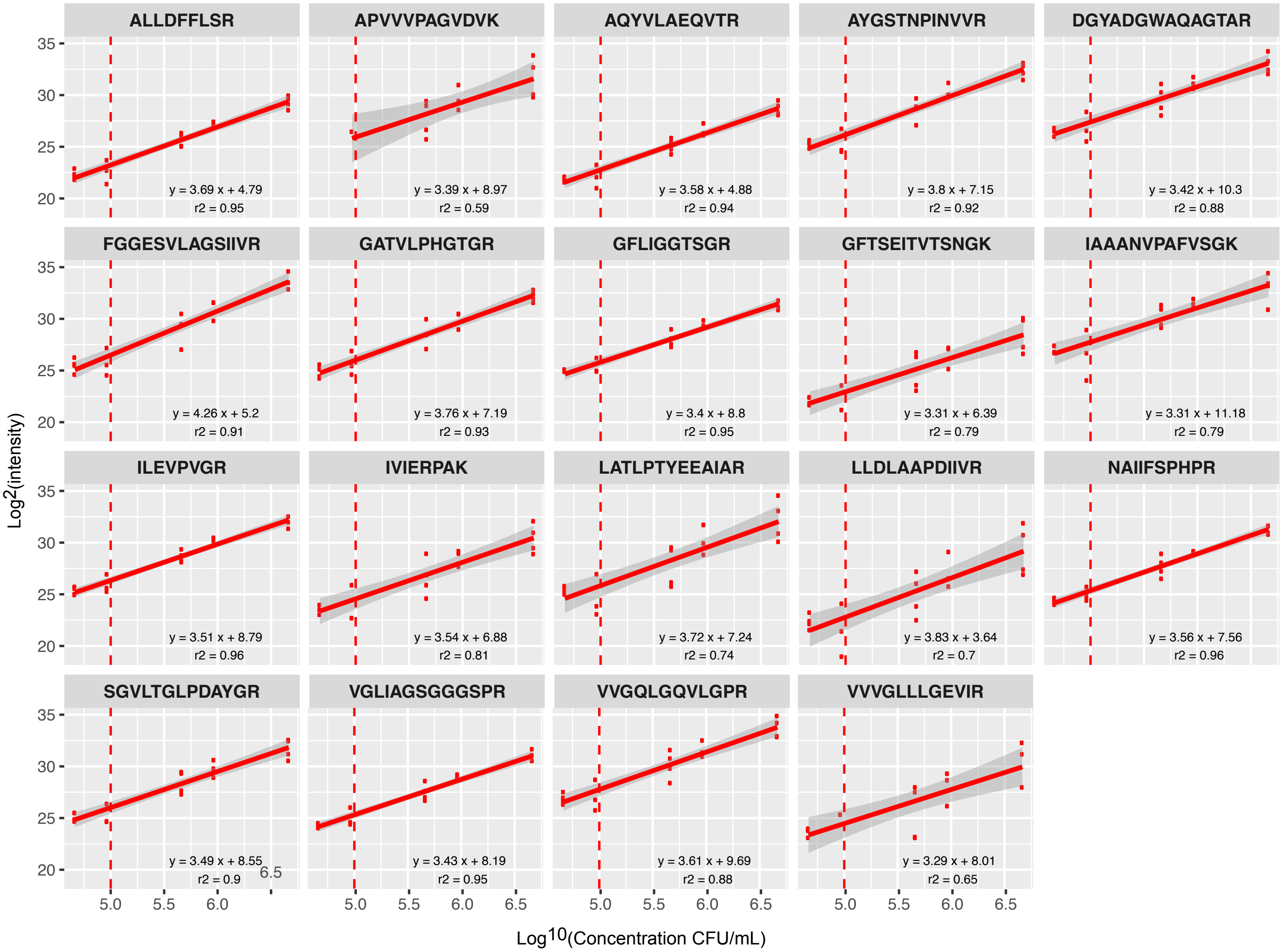

Supplementary Figure 8

b

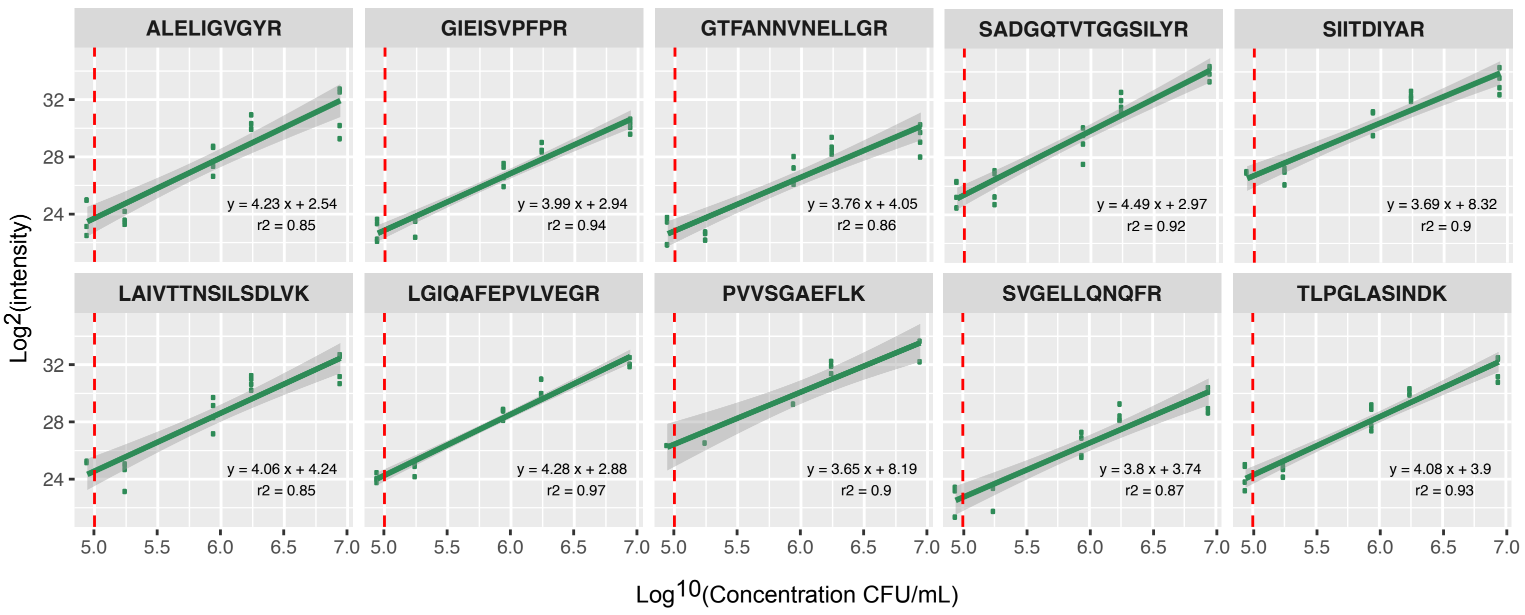

Supplementary Figure 8

c

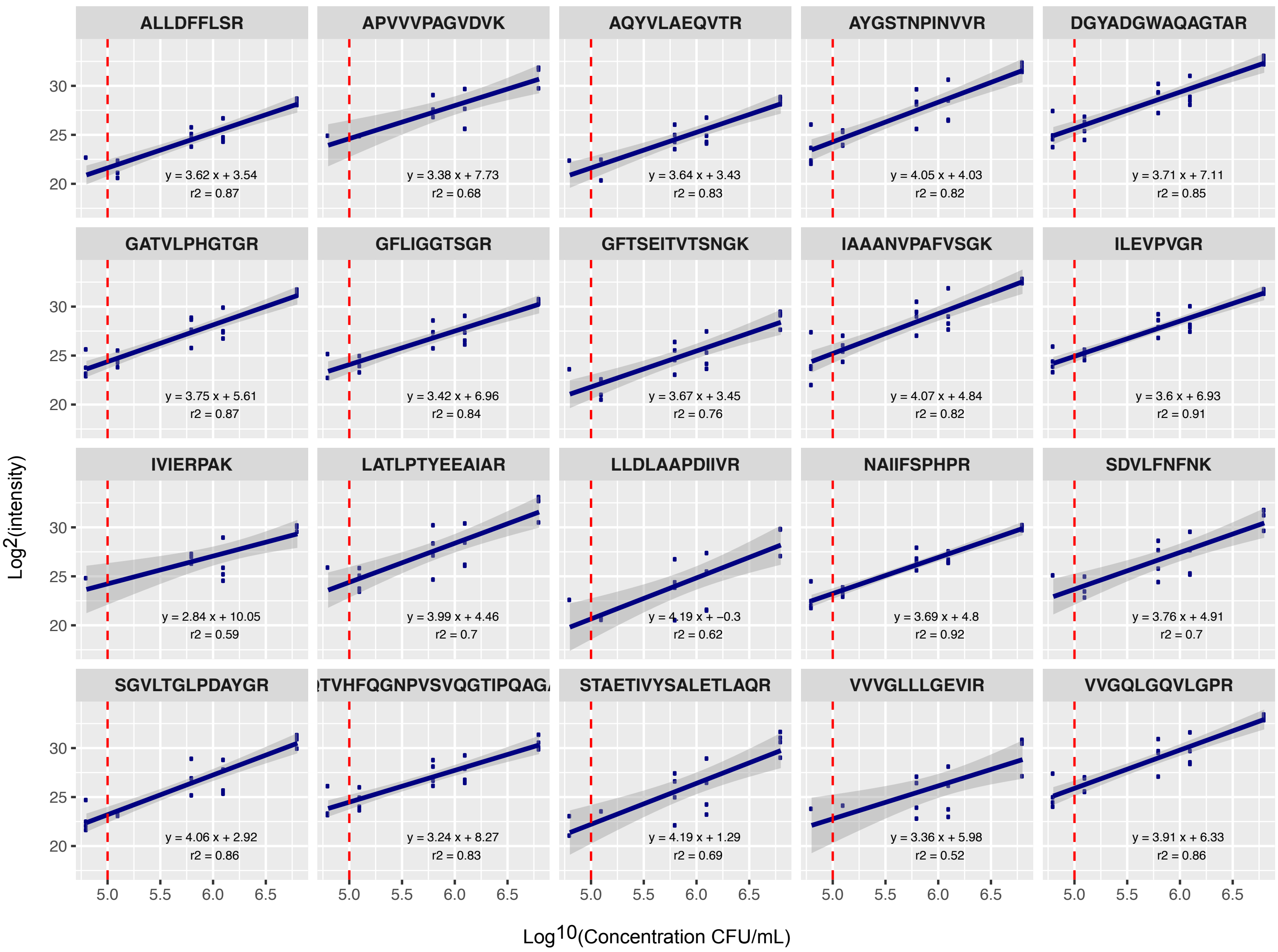

Supplementary Figure 8

d

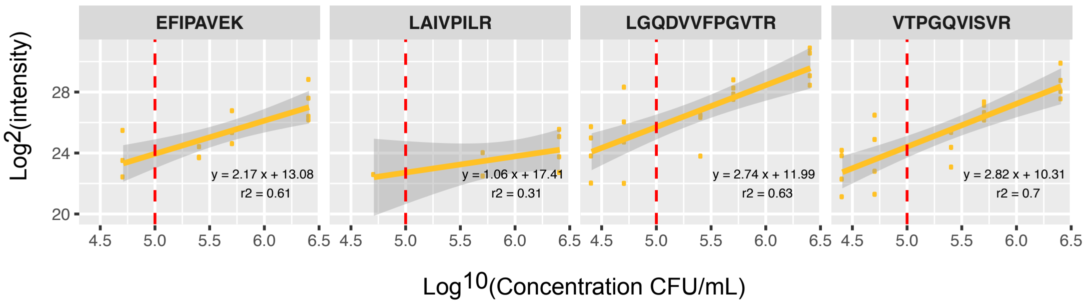

Supplementary Figure 9

a

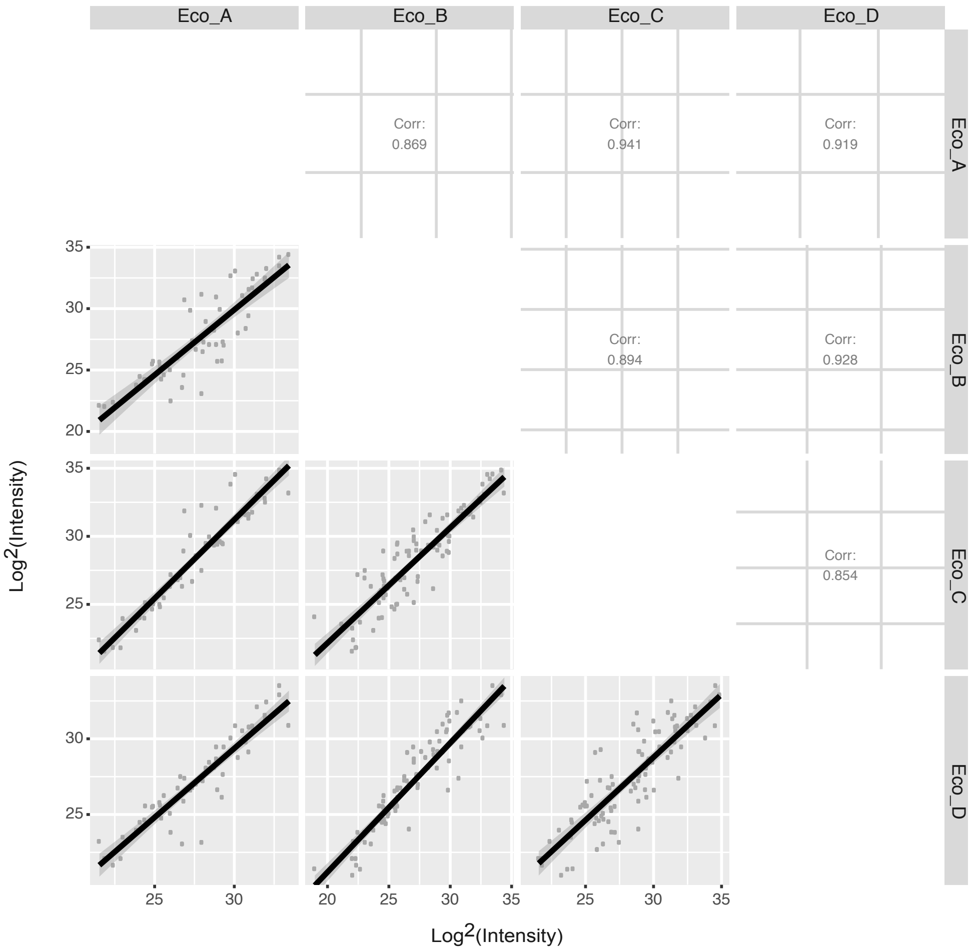

b

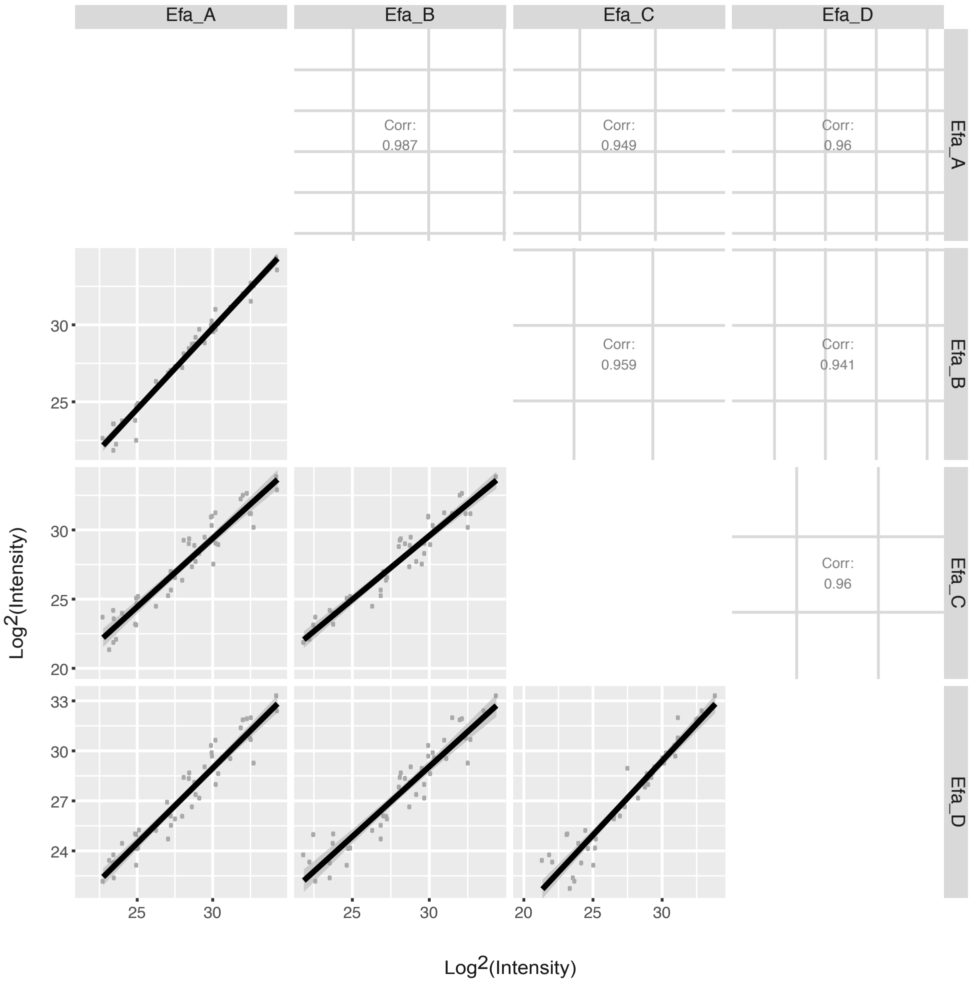

Supplementary Figure 9

c

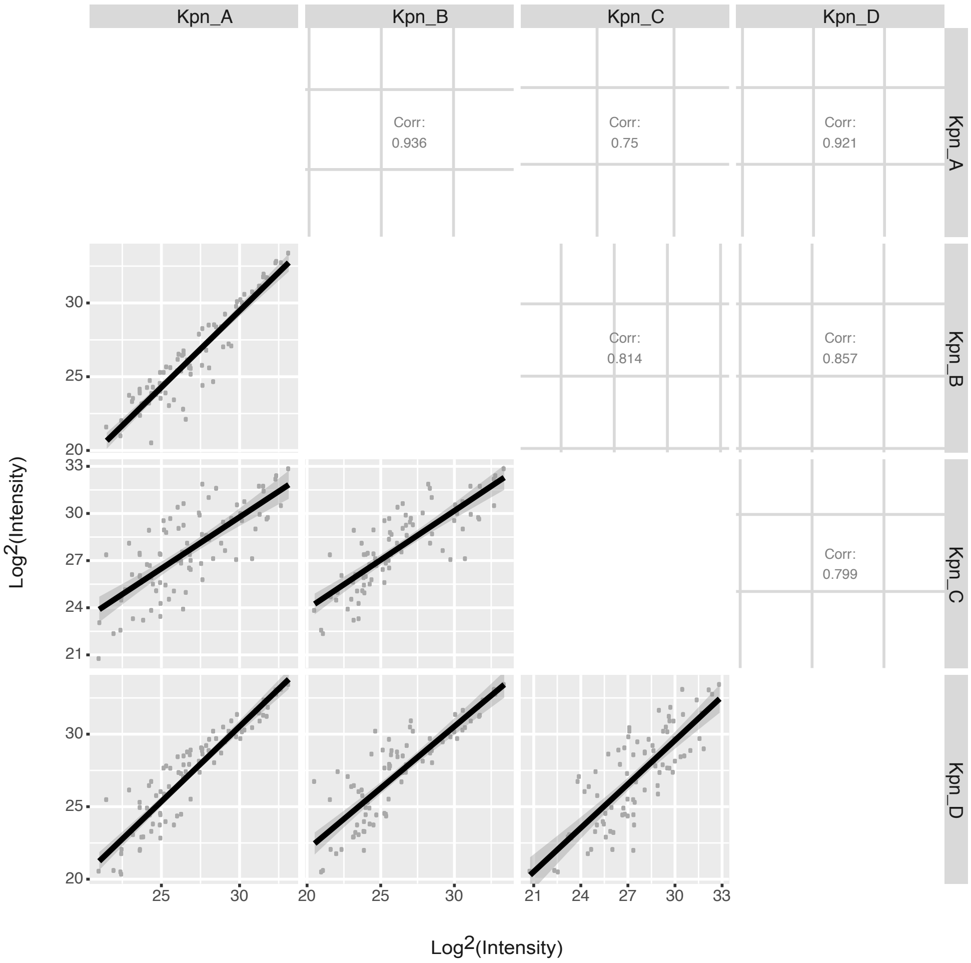

d

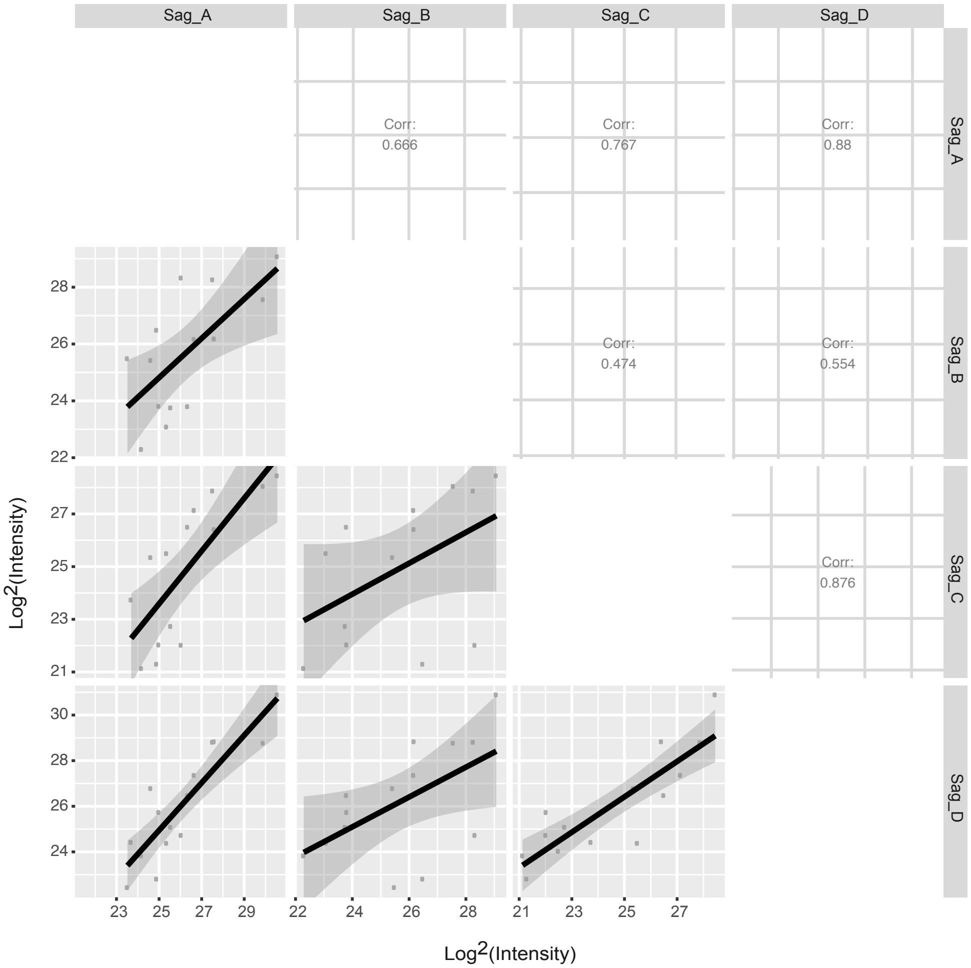

Supplement: Supp_Fig_II [file 152789_2_supp_402451_pyz8d4.pdf]
