# Supplementary material for: Fast and Accurate Bacterial Species Identification in Urine Specimens Using LC-MS/MS Mass Spectrometry and Machine Learning
Source: Mol Cell Proteomics. 2019 Oct 4;18(12):2492–505. doi: 10.1074/mcp.TIR119.001559 (PMC6885708; doi:10.1074/mcp.TIR119.001559)
Supplement: Supp_Meth [file 152789_2_supp_402452_pyr8d4.pdf]

Supplementary Methods

Bacterial Culture

Bacterial strains were obtained from the Culture Collection of Centre de Recherche en Infectiologie of Laval University (CCRI, Quebec, Canada) registered as WDCM861 at the World Data Centre for Microorganisms. Their identification numbers as well as their culture conditions are listed below:

Supplementary Methods Table 1:
Bacterial species and strains used

Abbreviation	Species	Strain	Culture condition	Gram-stain
Eco	*Escherichia coli*	CCRI-12923	aerobe	negative
Efa	*Enterococcus faecalis*	CCRI-95^T	aerobe	positive
Sag	*Streptococcus agalactiae*	CCRI-235^T	air+5%CO_2	positive
Kpn	*Klebsiella pneumoniae*	CCRI-563	aerobe	negative
Pae	*Pseudomonas aeruginosa*	CCRI-691	aerobe	negative
Sep	*Staphylococcus epidermidis*	CCRI-203	aerobe	positive
Sau	*Staphylococcus aureus*	CCRI-9443	aerobe	positive
Ssa	*Staphylococcus saprophyticus*	CCRI-223^T	aerobe	positive
Smi	*Streptococcus mitis*	CCRI-259^T	air+5%CO_2	positive
Sha	*Staphylococcus haemolyticus*	CCRI-210^T	aerobe	positive
Ecl	*Enterobacter cloacae*	CCRI-860	aerobe	negative
Kox	*Klebsiella oxytoca*	CCRI-1109	aerobe	negative
Cfr	*Citrobacter freundii*	CCRI-429^T	aerobe	negative
Pmi	*Proteus mirabilis*	CCRI-675	aerobe	negative
Kae	*Klebsiella aerogenes*	CCRI-446^T	aerobe	negative

T: denotes type strain. Identification of other strains was confirmed by gene sequencing and/or by MALDI-TOF

Each bacterial strain was plated on blood agar (TSA II 5% Sheep blood; Becton Dickinson) and incubated at 35°C overnight under aerobic conditions (or in air + 5% CO_2 for *S. agalactiae* and *S. mitis*). Bacterial colonies were then collected calibrated to a 0.5 MacFarland suspension in phosphate-buffered saline (PBS; 137mM NaCl, 6.4mM Na_2HPO_4, 2.7mM KCl, 0.88mM KH_2PO_4, pH 7.4). 30uL of this suspension were used to inoculate 3 mL of BHI medium (Brain Heart Infusion medium broth; Becton Dickinson) which was incubated overnight at 37°C under aerobic conditions with agitation (or 35°C in air + 5% CO_2 without agitation for *S. agalactiae* and *S. mitis*).
Finally, semi-log cultures were done by inoculation of 30µL of the previous culture in 3mL BHI medium and incubated in the same conditions. The cultures were stopped in exponential phase according to the previously known semi-log curve of each strain.

These cultures were used for spectral libraries generation or for urine inoculation. In parallel they were counted by incubation of 100uL of serial dilutions on Blood agar plates.

Supplementary Methods Table 2:
Uniprot protein databases for DDA analyses

**Organism**	**Taxon ID**	**Uniprot Proteome ID**	**Number of entries**	**Date**
Citrobacter freundii (Cfr)	546	UP000194801	5008	2017.07.06
Enterobacter cloacae (ECl)	550	UP000034104	4330	2018.05.09
Escherichia coli (Eco)	83333	UP000000625	4314	2016.03.15
Klebsiella aerogenes (Kae)	1028307	UP000008881	4909	2018.05.09
Klebsiella oxytoca (Kox)	571	UP000035545	6408	2017.07.06
Klebsiella pneumoniae (Kpn)	272620	UP000000265	5126	2017.07.06
Pseudomonas aeruginosa (Pae)	208964	UP000002438	5564	2017.05.12
Proteus mirabilis (Pmi)	584	UP000008319	3661	2018.05.09
Enterococcus faecalis (Efa)	226185	UP000001415	3240	2018.05.09
Streptococcus agalactiae (Sag)	208435	UP000000821	2105	2017.05.12
Staphylococccus aureus (Sau)	93061	UP000008816	2889	2017.06.26
Staphylococcus epidermidis (Sep)	176280	UP000001411	2461	2018.05.09
Staphylococcus haemolyticus (Sha)	279808	UP000000543	2640	2018.05.09
Streptococcus mitis (Smi)	28047	UP000027992	1983	2018.05.09
Staphylococcus saprophyticus (Ssa)	342451	UP000006371	2404	2018.05.09
Homo sapiens	9606	UP000005640	73928	2018.04.25

Supplementary Methods Table 3:
Mass Spectrometry parameters

	**DDA**	**DIA**	**PRM**	**PRM**
**Instrument**	Orbitrap Fusion	Orbitrap Fusion	Orbitrap Fusion	Q-Exactive H-FX
Method duration	120 min	120 min	120 min	50 min
Polarity	Positive	Positive	Positive	Positive
MS parameters				
Analyzer	Orbitrap	Orbitrap		
Resolution	120K	60K		
Mass Range	350-1800	400-1000		

AGC	4E+05	4E+05		
Max Injection time (ms)	50	50		
MSMS parameters				
Isolation window	1.6	10 (40 windows, 400-800)	0.7	0.7
Activation type	HCD	HCD	HCD	HCD
Collision energy	35%	35%	35%	28
Analyzer	Ion Trap	Orbitrap	Orbitrap	Orbitrap
Resolution or Scan rate	Rapid	30K	30K	15K
AGC	1E+04	4E+05	5E+04	1E+05
Max Injection time (ms)	50	70	120	100
Data Dependent MS2 parameters	Most intense precursor with intensity greater than 5000, Top speed 3s, Dynamic exclusion 20s with 10ppm tolerance.			
Internal calibration	Lock mass 445.12003	Lock mass 445.12003	Lock mass 445.12003	Lock mass 445.12003
