# Supplementary material for: Fast and Accurate Bacterial Species Identification in Urine Specimens Using LC-MS/MS Mass Spectrometry and Machine Learning
Source: Mol Cell Proteomics. 2019 Oct 4;18(12):2492–505. doi: 10.1074/mcp.TIR119.001559 (PMC6885708; doi:10.1074/mcp.TIR119.001559)
Supplement: Supp_Tab_1_7_8_10 [file 152789_2_supp_402453_pyl8xl.pdf]

Supplementary Table 1. **Summary of identification results obtained by LC-MSMS analysis of each pure bacterial species in DDA mode correlated with genomic data.** The table presents the number of proteins and peptides identified after database search of the MS data in Uniprot. The proteome coverage was calculated using the percentage of proteins identified regarding the number of entries in the database. The results were compared to the Total genomic length and Predicted protein count of each species found in NCBI genome database.

Supplementary Table 2. **Proteome Discoverer 2.1 identification results for each pure bacterial species analyzed by LC-MSMS in DDA mode**. Peptide export obtained after Mascot search and Percolator validation. Only high confidence peptides were kept. Each tab corresponds to one of the 15 bacteria of interest.

Supplementary Table 3. **Result of Unipept search.** For each of the 31096 peptides tested, Unipept reports if its sequence can be found (1) or not (0) in each of 15 bacteria of interest.

Supplementary Table 4. **List of peptides identified and quantified by DIA**. List of peptides selected from DIA analyses of inoculated urine with each of the 15 bacterial species (9 high-level, 3low-level replicates) and their quantification values. The DIA signal was extracted using the Skyline software and the spectral libraries previously generated on pure bacterial cultures. Criteria for peptide identification and how quantification was performed in Skyline is reported in the Experimental Procedures section.

Supplementary Table 5. **Peptides signatures reported by machine learning classifiers.** List of the peptide signatures reported by each of the 3 machine learning models retained in our study (Hoeffding Tree, NaiveNayes and BayesNet), list of the merged signature and final 82 peptide signature after manual curation.

Supplementary Table 6. **Transitions List of the 82 peptides signature.** List of precursor masses targeted in PRM experiments and corresponding fragments used for quantification of these peptides in Skyline.

Supplementary Table 7. **Count of bacterial cultures reported to urine inoculation.** Bacterial cultures were counted as described in the Methods section and the values obtained were used to determine the bacterial concentration in urine inoculates.

Supplementary Table 8. **Filtering criteria applied on the known PRM datasets after Skyline extraction and their associated wrong prediction rate.** Different combination of three criteria - Skyline library dot-product (dotP), mass error (in ppm) or q-value derived from second best peaks scoring - were attempted and set to different values.

Supplementary Table 9. **Probabilities of bacterial contamination of inoculated urine and patient specimens.** Probabilities associated to bacterial identification reported by the

prediction model after PRM monitoring of the peptidic signature on inoculated samples analyzed on (a) Orbitrap Fusion, 90 minutes gradients; (b) Q-Exactive, 30 minutes gradients or on (c) patient specimens.

Supplementary Table 10. **Bacterial identification reported by MALDI-TOF and LC-MSMS analyses.** For samples analyzed with both methods, the “MALDI-TOF” column shows the results obtained with the standard procedure at Enfant-Jesus Hospital of Quebec City, Canada, the “LC-MS” column shows the prediction given by the algorithm with our method.

Supplementary Table 1

Organism	Gram	Taxon ID	Uniprot database Entries	Nbr Proteins	Nbr Protein Groups	Nbr Modified Peptide Groups	Proteome Coverage (%)	Total Genomic Length (Mb)	Predicted Protein Count
Citrobacter freundii (Cfr)	-	546	5008	1806	1797	25705	36.1	5.27	4985
Enterobacter cloacae (Ecl)	-	550	4330	1134	1121	11407	26.2	4.93	4613
Escherichia coli (Eco)	-	83333	4314	1787	1772	24726	41.4	5.15	5009
Klebsiella aerogenes (Kae)	-	1028307	4909	1096	1085	10686	22.3	5.24	4915
Klebsiella oxytoca (Kox)	-	571	6408	1616	1583	17206	25.2	6.04	5608
Klebsiella pneumoniae (Kpn)	-	272620	5126	1914	1899	26888	37.3	5.59	5407
Pseudomonas aeruginosa (Pae)	-	208964	5564	2453	2438	29558	44.1	6.6	6103
Proteus mirabilis (Pmi)	-	584	3661	1307	1300	14913	35.7	3.95	3500
Enterococcus faecalis (Efa)	+	226185	3240	1128	1123	16986	34.8	2.99	2919
Streptococcus agalactiae (Sag)	+	208435	2105	867	863	13101	41.2	2.08	2015
Staphylococcus aureus (Sau)	+	93061	2889	1146	1142	15902	39.7	2.84	2820
Staphylococcus epidermidis (Sep)	+	176280	2461	1144	1140	13110	46.5	2.53	2373
Staphylococcus haemolyticus (Sha)	+	279808	2640	986	986	10987	37.3	2.5	2349
Streptococcus mitis (Smi)	+	28037	1983	810	810	11411	40.8	1.98	1838
Staphylococcus saprophyticus (Ssa)	+	342451	2404	1163	1161	14970	48.4	2.59	2401
Total number of Peptide Groups						257556			
Number of Non-Redundant Stripped Sequences						126859			
Number of Non-Redundant Stripped Sequences after filtering						31096			

Supplementary Table 7

Sample Name	Bacterial culture concentration (CFU/mL)	Inoculated volume (µL)	Final concentration in urine (CFU/mL)
For validation by targeted proteomics			
UP 1	4.63E+08	1	4.63E+04
UP 2	4.63E+08	2	9.26E+04
UP 10	4.63E+08	10	4.63E+05
UP 20	4.63E+08	20	9.26E+05
UP 100	4.63E+08	100	4.63E+06
EF 1	8.77E+08	1	8.77E+04
EF 2	8.77E+08	2	1.75E+05
EF 10	8.77E+08	10	8.77E+05
EF 20	8.77E+08	20	1.75E+06
EF 100	8.77E+08	100	8.77E+06
KP 1	6.30E+08	1	6.30E+04
KP 2	6.30E+08	2	1.26E+05
KP 10	6.30E+08	10	6.30E+05
KP 20	6.30E+08	20	1.26E+06
KP 100	6.30E+08	100	6.30E+06
SA 2	1.28E+08	2	2.56E+04
SA 4	1.28E+08	4	5.12E+04
SA 20	1.28E+08	20	2.56E+05
SA 40	1.28E+08	40	5.12E+05
SA 200	1.28E+08	200	2.56E+06
For comparison with MALDI-TOF			
Spike_Sag	1.17E+08	100	1.17E+06
Spike_Pae	2.83E+08	200	5.66E+06
Spike_Pmi	N/A	20	N/A
Spike_Ecl	3.20E+08	20	6.40E+05
Spike_Kox	8.37E+08	20	1.67E+06
Spike_Cfr	8.40E+08	20	1.68E+06
Spike_Kae	9.30E+08	20	1.86E+06
Spike_Sm	3.97E+08	20	7.94E+05
Spike_Ecc	3.03E+08	20	6.06E+05
Spike_Efa	8.07E+08	20	1.61E+06
Spike_Kpr	6.07E+08	20	1.21E+06
Spike_Sep	1.17E+08	200	2.34E+06
Spike_Ssa	1.93E+08	20	3.86E+05
Spike_Sau	2.26E+08	100	2.26E+06
Spike_Sha	1.66E+08	20	3.32E+05

Supplementary Table 8

**Filtering criteria**	**Wrong prediction rate**
dotP > 0.85 & ppm < 10 OR dotP > 0.75 & ppm < 3	1,20%
q-value < 0.01	4,82%
q-value < 0.05	2,41%
dotP < 0.75 & *q*-value < 0.01	5,42%
dotP < 0.85 & *q*-value < 0.01	9,04%
dotP > 0.85 & ppm < 10 & *q*-value < 0.01 OR dotP > 0.75 & ppm < 3 & *q*-value < 0.01	6,63%
dotP > 0.85 & ppm < 10 & *q*-value < 0.05 OR dotP > 0.75 & ppm < 3 & *q*-value < 0.05	1,81%

Supplementary Table 10

Sample Name	MALDI-TOF	LC-MS
Patient urine specimens		
Patient_038	Escherichia coli	Eco
Patient_C01	Absence of uropathogens	Blk
Patient_C04	Staphylococcus saprophyticus	Ssa
Patient_C06	Absence of uropathogens	Blk
Patient_C07	Streptococcus epidermidis	Sep
Patient_C08	Absence of uropathogens	Blk
Patient_C10	Absence of uropathogens	Blk
Patient_C11	Escherichia coli	Eco
Patient_C12	Absence of uropathogens	Blk
Patient_C14	Streptococcus probable	Blk
Patient_D02	Enterococcus problable	Blk
Patient_D03	Enterococcus problable	Blk
Patient_D05	Escherichia coli	Eco
Patient_D10	Streptococcus agalactiae	Blk
Patient_D14	Escherichia coli	Eco
Patient_D21	Escherichia coli	Eco
Patient_D22	Staphylococcus probable	Blk
Patient_D26	Staphylococcus probable	Blk
Patient_D31	Escherichia coli	Eco
Patient_D32	Staphylococcus probable	Blk
Patient_D40	Klebsiella pneumoniae	Kpn
Patient_D47	Escherichia coli	Kae
Patient_E01	Absence of uropathogens	Blk
Patient_E05	Absence of uropathogens	Blk
Patient_E08	Absence of uropathogens	Blk
Patient_E09	Escherichia coli	Eco
Patient_E30	Escherichia coli	Eco
Patient_E34	Enterococcus problable	Blk
Patient_E39	Enterococcus faecalis	Efa
Patient_E42	Escherichia coli	Eco
Patient_E48	Escherichia coli	Eco
Bacterial inoculation in urine		
Spike_Cfr	Citrobacter freundii	Cfr
Spike_Ecl	Enterococcus cloacae complex	Ecl
Spike_Eco	Escherichia coli	Eco
Spike_Efa	Enterococcus faecalis	Efa
Spike_Kae	Enterococcus cloacae complex	Kae
Spike_Kox	Klebsiella oxytoca	Kox
Spike_Kpn	Klebsiella pneumoniae	Kpn
Spike_Pae	Pseudomonas aeruginosa	Pae
Spike_Pmi	Proteus mirabilis	Pmi
Spike_Sag	Streptococcus agalactiae	Sag
Spike_Sau	Staphylococcus aureus	Sau
Spike_Sep	Streptococcus epidermidis	Sep
Spike_Sha	Staphylococcus haemolyticus	Sha
Spike_Smi	Candida albicans	Smi
Spike_Ssa	Staphylococcus saprophyticus	Ssa
